# Da‐Bu‐Yin‐Wan and Qian‐Zheng‐San Alleviate Parkinson's Disease by Activating the Keap1/Nrf2/HO‐1 Pathway to Positively Regulate Oxidative Stress

**DOI:** 10.1002/cns.70681

**Published:** 2025-12-09

**Authors:** Huimin Zhu, Zijian Liu, Jing Feng, Die Hu, Xia Li, Zhenyu Guo, Xueying Zhu, Cong Gai

**Affiliations:** ^1^ School of Traditional Chinese Medicine Beijing University of Chinese Medicine Beijing China; ^2^ Graduate School of the First Clinical Medical College Beijing University of Chinese Medicine Beijing China

**Keywords:** apoptosis, Bu‐yin‐Qian‐Zheng formula, Keap1/Nrf2/HO‐1 pathway, oxidative stress, Parkinson's disease

## Abstract

**Background:**

Parkinson's disease (PD) is a neurodegenerative disorder that worsens progressively. Different pathological mechanisms are involved in the progression of PD, including the loss of dopaminergic (DA) neurons and exacerbated oxidative damage in the nigrostriatal path. Da‐Bu‐Yin‐Wan combined with Qian‐Zheng‐San is called Bu‐Yin‐Qian‐Zheng Formula (BYQZF), which is a prescription for PD used in traditional Chinese medicine, but its neuroprotective effects and mechanisms are not well understood.

**Purpose:**

The purpose of this study was to explore how BYQZF can activate the Keap1/Nrf2/HO‐1 pathway to effectively lessen oxidative damage, providing insights into its potential anti‐PD effects.

**Methods:**

The chemical constituents and pharmacokinetics of BYQZF were analyzed by ultraperformance liquid chromatography in tandem with mass spectrometry (UPLC‐MS/MS). Then, we evaluated the toxicity and safety of BYQZF treatment. After establishing MPTP‐induced PD mouse models and the administration of BYQZF treatment, PD‐like behaviors were assessed through the pole test, rotarod test and open field experiment. DA neuron loss and apoptosis in the nigrostriatal path were detected via immunofluorescence and western blotting analysis. Oxidative stress levels and the dissociation state of Keap1‐Nrf2 in these brain areas were assessed by ELISA, immunofluorescence Co‐IP, RT‐qPCR, and western blot analysis. We constructed Nrf2‐knockdown PD cell models. The transcriptional and Nrf2 protein expression levels were determined using both real‐time quantitative PCR and western blot analysis. We further assessed cellular viability, intracellular ATP content, apoptotic cell ratio, as well as the expression levels of CAT and GSH following MPP^+^, and BYQZF exposure.

**Results:**

The chemical constituents of BYQZF were systematically characterized using UPLC‐MS/MS technology. The results of pharmacokinetic evaluation suggested that the pathological conditions of PD significantly affected the pharmacokinetic behavior of BYQZF in vivo. BYQZF treatment ultimately improved the movement disorders in MPTP‐induced PD mice, by alleviating the damage of dopaminergic neurons within the nigrostriatal path. In addition, BYQZF reduced the damage caused by oxidative stress by decreasing the 8‐OHdG level and increasing the CAT, GSH/GSSG, HO‐1, NQO1, and GCLM levels. Further, BYQZF treatment advanced the dissociation of Keap1 and Nrf2 and the nuclear translocation of Nrf2. Finally, functional experiments by Nrf2‐knockdown PD cell models demonstrated that the neuroprotective effect of BYQZF depends on the integrity of Nrf2, and Nrf2 deficiency significantly weakened its antioxidant and anti‐apoptotic effects.

**Conclusion:**

This study elucidated that BYQZF improved PD through the Keap1/Nrf2/HO‐1 signaling pathway and alleviated oxidative stress, which is beneficial for inspiring new strategies for drug development targeting PD.

## Graphical abstract

BYQZF treatment alleviated the oxidative stress damage through which BYQZF promoted the downstream antioxidant enzyme expressions of CAT, GSH/GSSG, NQO1, HO‐1, GCLM, and reduced the levels of 8‐OHdG detected in the plasma and midbrain. BYQZF treatment promoted the uncoupling of Keap1 and Nrf2 and the transfer of Nrf2 to the nucleus. This study elucidated that BYQZF improved PD through the Keap1/Nrf2/HO‐1 signaling axis and alleviated oxidative stress, which is beneficial for inspiring new ideas for creating drugs aimed at treating PD.

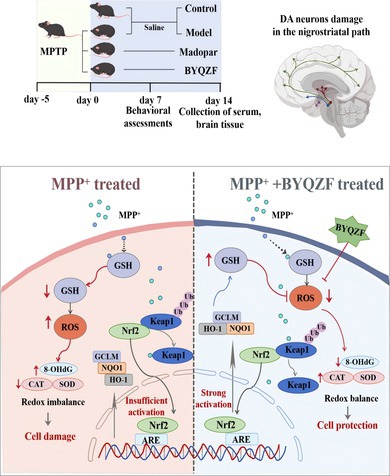

## 1 Introduction

Parkinson's disease is a neurodegenerative disorder that commonly affects middle‐aged and elderly individuals [1]. Epidemiological research indicates that Parkinson's disease has an effect on approximately 1% of the population aged over 60 in European and American populations, with prevalence rising to more than 4% among those aged over 80 [2]. Its pathological features primarily involve progressive diminishment of dopamine‐producing neurons along the nigrostriatal path, accompanied by the abnormal accumulation of Lewy bodies (intracellular inclusions containing α‐synuclein with atypical folding, typically appearing as fibrous aggregates), along with a reduction in dopamine neurotransmitters in the striatum and an imbalance between dopamine and acetylcholine neurotransmitters [3, 4]. The clinical symptoms include motor symptoms (for instance: bradykinesia, rigidity, tremors, and postural balance disturbances) and non‐motor symptoms (autonomic dysfunction, depression, anxiety, cognitive deficits, etc.) [5, 6]. The onset of PD motor symptoms can be attributed to the progressive degeneration of DA neurons, which leads to a significant reduction in dopamine signaling from the substantia nigra (SN) to the striatum, disrupting the dynamic equilibrium of neural circuits in the basal ganglia [7, 8]. Notably, oxidative stress, a key pathological mechanism, plays an important part in PD development [8].

Oxidative stress refers to the ROS and other free radicals at a rate exceeding the clearing capacity of the body's protective antioxidant processes (such as superoxide dismutase, catalase, glutathione, etc.), leading to a series of molecular damages, including lipid peroxidation, DNA strand breaks, mitochondrial dysfunction, and protein misfolding, ultimately inducing cell apoptosis or necrosis [9].

In the pathogenesis of PD, DA neurons in the SN are particularly sensitive to oxidative stress, mainly due to their physiological structure and metabolic characteristics: on one hand, these neurons have highly branched axonal trees and a high basal metabolic rate, making them highly dependent on energy supply and demand balance [10]; on the other hand, dopamine, as a molecule prone to auto‐oxidation, generates a significant amount of ROS and quinone intermediates during metabolism, which are directly toxic to cells [11]. In this context, mitochondrial dysfunction (especially reduced activity of complex I), abnormal aggregation of α‐synuclein, and the decline in proteasomal degradation further exacerbate oxidative stress, creating a vicious cycle with lipid peroxidation, DNA damage, and other pathological mechanisms, further worsening neuronal damage [12, 13]. Consequently, oxidative stress is a major factor in driving the degenerative changes in dopaminergic neurons in PD. Understanding the temporal role of oxidative stress, key molecular targets, and its integration with other pathological mechanisms is crucial for identifying more targeted intervention strategies.

The Nrf2/Keap1/HO‐1 pathway, as a key regulatory pathway for improving oxidative stress and environmental stress, has attracted widespread interest among researchers [14]. The stability and transcriptional activity of Nrf2 have been shown to be closely related to the initiation of antioxidant defense mechanisms, and are regulated by histone modifications, microRNAs, DNA methylation, and specific amino acid chemical modifications [15]. Studies have demonstrated that Nrf2 influences the expression of a large number of genes encoding redox‐regulating factors and stress‐response proteins [16]. In typical circumstances, Nrf2 is constantly degraded by Keap1 to maintain low‐level expression [17, 18].

Under cellular stress conditions—such as oxidative stress, impaired autophagy, or endoplasmic reticulum stress—the key cysteine residues of Keap1 (e.g., Cys151 and Cys273) undergo oxidative modifications. These changes alter the DGR domain of Keap1, weakening its binding affinity for the DLG motif in Nrf2. As a result, Nrf2 evades ubiquitin‐mediated proteasomal degradation, gathers in the cytoplasm before transferring to the nucleus [19, 20]. Hence, Nrf2 functions as a detector for cellular responses to stress signals such as autophagy defects and DNA damage [19]. Once inside the nucleus, Nrf2 pairs with small Maf proteins to form a heterodimer and connects with antioxidant response elements, triggering the expression of antioxidant and cell‐protecting genes, including NQO1, GPx, HO‐1, and SOD, thereby enhancing the cell's ability to react to stress [19, 20].

Currently, therapeutic choices for PD include levodopa, dopamine receptor agonists, and anticholinergic drugs [6, 21], with surgical treatments mainly including deep brain stimulation and destructive surgeries such as pallidotomy and thalamotomy [6]. Patients treated with dopamine replacement drugs may develop resistance to levodopa, leading to a reduction in efficacy or causing motor fluctuations (such as the “on–off phenomenon”), where symptoms suddenly improve or worsen, becoming difficult to control [21]. Dopamine receptor agonists can cause harmful reactions like somnolence, impulse control issues and hallucinations, and are typically less effective than levodopa [6, 21]. Electrode implantation may be associated with issues like infection, bleeding, and electrode displacement, while destructive surgeries may lead to cognitive impairments, emotional issues, and other neurological complications [6].

Currently, several small‐molecule drugs such as Epalrestat and dimethyl fumarate have been developed as Nrf2 activators, demonstrating modest efficacy in clinical or animal studies. However, except for dimethyl fumarate which has a relatively established clinical approval profile, most candidate molecules remain at the preclinical or animal model stage, with no large‐scale clinical trial data available for PD patients. For the majority of therapeutics targeting the Keap1/Nrf2/HO‐1 axis, well‐established evidence regarding long‐term efficacy, safety, and population applicability remains lacking [22]. Furthermore, some existing Nrf2 activators face challenges in prolonged applications—including excessive activation effects, interference with other metabolic pathways, or potential tolerance induction [23]. Critically, single‐target Nrf2 activators typically exhibit monolithic mechanisms of action, failing to address PD's multifaceted pathological processes such as neuroinflammation, neuronal apoptosis, and mitochondrial dysfunction.

Given the limitations and adverse effects of existing treatments, increasing attention is being given to complementary and alternative treatments, including traditional Chinese medicine (TCM). Bu‐Yin‐Qian‐Zheng Formula (BYQZF), a traditional compound formulation composed of Da‐Bu‐Yin‐Wan (DBYW, *Great Yin Tonic Pill*) and Qian‐Zheng‐San (QZS, *Symmetry Leading Powder*). The formula includes key herbs such as *Anemarrhenae rhizoma* (Zhi‐Mu), Rehmanniae *Radix praeparata* (Shu‐Di‐Huang), *Testudinis plastrum* (Gui‐Ban), *Phellodendri chinensis cortex* (Huang‐Bai), Prepared Giant *Typhonii rhizoma* (Zhi‐Bai‐Fu‐Zi), Scorpio (Quan‐Xie), and *Bombyx batryticatus* (Jiang‐Can). BYQZF is derived from clinical experience formulas, and it may have potential advantages in improving multisystem symptoms (motor disorders, non‐motor symptoms) of PD by targeting the mechanism of nourishing yin and tonifying kidney, suppressing hyperactive liver and subsiding yang, dispelling wind and resolving phlegm, rather than just antioxidant effects. Its multitarget characteristics may help overcome resistance or side effects. Compared to single‐target drugs, BYQZF exerts the effect of reducing toxicity and increasing efficiency through multicomponent, multitarget, and multipathway synergistic actions. It has been widely applied in clinical TCM treatments for PD [24, 25]. Our previous research has shown that DBYW and QZS synergistically possess anti‐parkinsonism and neuroprotective properties; the combined formula (BYQZF) is more effective [26]. Our previous study also demonstrated that BYQZF had multiple neuroprotective benefits against PD, including improving mitochondrial function, ameliorating oxidative stress damage, alleviating motor dysfunction and so on [27, 28, 29, 30, 31, 32]. However, its specific mechanisms, particularly its regulatory effects on the Nrf2/Keap1/HO‐1 antioxidant pathway, have not been completely clarified. This study expands the understanding of the mechanisms underlying BYQZF's effects, providing new experimental evidence for its modernized and precise application in PD treatment.

## 2 Materials and Methods

### 2.1 Animals Used in the Experiment

Six‐week‐old male C57BL/6J mice, with an average weight of 20 ± 2 g, were procured from Spelford (Beijing) Biotechnology Co. Ltd. All animal experiments were carried out in accordance with the “Guidelines for Care and Use of Laboratory Animals” (NIH Publication No. 85‐23, revised 1996) published by the US National Institutes of Health. All procedures were approved by the Animal Care Committee of Beijing University of Chinese Medicine (Approval Code: BUCM‐2024051602‐2130).

### 2.2 Chemicals and Reagents

The following instruments and materials were used in this experiment. The SH‐SY5Y cell line, derived from human neuroblastoma, was acquired from Shanghai GK Gene Chemical Co. Ltd. The behavioral testing equipment for animals, including the Rotarod Apparatus and open field experiment box, was purchased from San Diego Instruments (USA), while the climbing rod device was custom‐made in the laboratory.

1‐Methyl‐4‐phenylpyridinium (MPP^+^) used for cell injury models, MPTP hydrochloride used for the PD mice models, and Levodopa/Benserazide (Medopa) were all purchased from Roche, USA. The ATP detection kit and oxidized glutathione (GSSG) detection kit were purchased from Beyotime, China. The Annexin V‐PE apoptosis detection kit used for cell apoptosis analysis was purchased from KeyGEN BioTECH, China. The TUNEL staining kit was purchased from Roche Diagnostics (Germany). Protein concentration was determined using the BCA assay kit, and chemiluminescence detection for Western blotting was carried out using chemiluminescence methods. All of these were purchased from Thermo Fisher, USA. The electroporation device for cells was also purchased from Thermo Fisher Scientific, USA. Antibodies for Western blotting and immunofluorescence staining (Bax, TH, Keap1, caspase‐3, Nrf2, Bcl‐2, GAPDH, etc.) were purchased from Abcam plc (Cambridge, UK) or Cell Signaling Technology (CST, USA). ELISA kits were purchased from Elabscience, and qPCR‐related reagents (RNA extraction kits, reverse transcription kits, qRT‐PCR reaction enzymes, primers) were obtained from Applied Biosystems.

### 2.3 Drug Preparation and Identification

The Bu‐Yin‐Qian‐Zheng Formula is composed of seven medicinal herbs (Table 1). All raw materials were purchased from a GMP‐certified supplier, Beijing Tongrentang Traditional Chinese Medicine Decoction Pieces Co. Ltd., and were accurately weighed according to the standard and compound ratios specified in the 2020 edition of the Pharmacopeia of the People's Republic of China. The mixed herbs were soaked in 8 times their volume of distilled water for half an hour and then simmered on two occasions, each over the course of 1 hThe two decoctions were mixed together, filtered, and adjusted to a concentration of 0.5 g/mL, membrane‐filtered, separated into aliquots, and stored at −80°C.

**TABLE 1:** Component herbs of BYQZF.

Chinese name	Pharmacopeia	Common name	Weight (g)
Shu‐Di‐Huang	*Rehmanniae Radix Praeparata*	Prepared rehmannia root	36
Gui‐Ban	*Testudinis Plastrum*	Tortoise plastron	36
Zhi‐Mu	*Anemarrhenae Rhizoma*	Common anemarrhena	24
Huang‐Bai	*Phellodendron chinense* C.K. Schneid.	Amur cork tree bark	24
Jiang‐Can	*Bombyx Batryticatus*	Stiff silkworm	24
Zhi‐Bai‐Fu‐Zi	Prepared Giant *Typhonium Rhizome*	Giant typhonium tuber	24
Quan‐Xie	Scorpio	Chinese scorpion (detoxicated)	24

### 2.4 UPLC‐MS/MS Analysis of BYQZF

An Acquity UPLC I‐Class system (Waters, MA, USA) with an online degassing system, autosampler, column temperature chamber, and UV detector was employed. Chromatography was performed under these conditions: Chromatographic column: Acquity UPLC HSS T3 (100 mm × 2.1 mm, 1.8 μm) (Waters, MA, USA); column temperature: 45°C; mobile phase: A‐ 0.1% phosphoric acid solution (Thermo Fisher, USA), B‐ chromatographic acetonitrile (Thermo Fisher, USA); flow rate: 0.35 mL/min; injection volume: 2 μL; PDA detection wavelength: 210–400 nm. The conditions for UPLC elution were set as follows: 0–2 min: 95%–95% A, 5%–5% B; 2–4 min: 95%–70% A, 5%–30% B; 4–8 min: 70%–50% A, 30%–50% B; 8–10 min: 50%–20% A, 50%–80% B; 10–14 min: 20%–0% A, 80%–100% B; 14–15 min: 0–0A, 100%–100% B; 15–15.1 min: 0%–95% A, 100%–5% B; 15.1–16 min: 95%–95% A, 5%–5% B.

The components of BYQZF were further analyzed by ultraperformance liquid chromatography in tandem with mass spectrometry (UPLC‐MS/MS) (Thermo Fisher, USA). Thermo‐Orbitrap‐QE HF mass spectrometer was utilized with positive and negative HESI ionization sources. The mass spectrometry parameters are detailed in Table 2.

**TABLE 2:** The mass spectrometry parameters.

Parameters	Positive	Negative
Spray voltage (V)	3800	−3200
Capillary temperature (°C)	320	320
Aux gas heater temperature (°C)	350	350
Sheath gas flow rate (Arb)	35	35
Aux gas flow rate (Arb)	8	8
S‐lens RF level	50	50
Mass range (m/z)	100–1500	100–1500
Full ms resolution	60,000	60,000
MS/MS resolution	15,000	15,000
NCE/stepped NCE	10, 20, 40	10, 20, 40

### 2.5 Animal Models, Treatment, and Behavioral Evaluation

Six‐week‐old male C57BL/6J mice (20 ± 2 g) in the control group received saline gavage for 5 days. Mice in the model, Madopar, and BYQZF groups were injected with 20 mg/kg MPTP daily for 5 days to induce PD. BYQZF doses were calculated using the formula “dosage = (human daily dose ÷ 60 kg) × 6.25 × 4.” Mice in the BYQZF group received oral BYQZF for 14 days (low: 5 g/kg, 10 mL/kg, 0.5 g/mL; high: 15 g/kg, 10 mL/kg, 1.5 g/mL), while the Madopar group received 62.5 mg/kg for 14 days. Behavioral tests were performed on day 7, and serum and brain tissues were collected on day 15. Motor coordination was assessed using the rotarod test, where mice were placed on a rotating rod (3–4 cm diameter, 50 cm length) with speed increasing from 4 rpm by 1–2 rpm per minute until fall. Each mouse underwent 3–5 trials with 10–15 min intervals, recording latency to fall and number of falls. Bradykinesia was evaluated using the pole test, measuring the time to turn and descend within 120 s over three trials, with the average used as the result. Spontaneous motor activity and anxiety‐like behavior were assessed in a 40 × 40 × 20 cm open field divided into 16 squares. Mice were placed at the center, and movement trajectories, cumulative distance, average speed, and central area entries were recorded over 10 min. Equipment was cleaned between trials, and lighting and noise were kept consistent.

### 2.6 Toxicity and Safety Evaluation of BYQZF

In order to evaluate the toxicity and safety of BYQZF, we also set up two additional mice groups, low concentration BYQZF (5 g crude drug/kg) treatment without MPTP and high concentration BYQZF (15 g crude drug/kg) treatment without MPTP for 14 days. At the end of the 14‐d experimental period, we collected vital organs, including the heart, liver, spleen, lung, kidney, stomach, intestines, and brain, and performed H&E staining. We also collected the supernatant from blood extraction for serum biochemical analysis and inflammatory factors detection by ELISA kits.

### 2.7 Pharmacokinetic Studies

6‐week‐old male C57BL/6J mice (20 ± 2 g; *n* = 6 per group) were orally administered the herbal decoction (BYQZF) at a dose of 5 g crude drug/kg. The administration volume was 10 mL/kg, and the concentration of the drug solution was 0.5 g/mL. Plasma samples were collected on day 14 after 14 consecutive days of drug administration. Plasma samples harvested via the ophthalmic venous plexus were collected at 0.5, 1, 2, 4, 6, 8, 12 and 24 h post‐administration. The supernatant samples were collected after centrifugation (5000 rpm, 10 min) and stored at −80°C immediately until analysis.

Each 100 μL plasma aliquot obtained at predetermined intervals was supplemented with 5 μL of internal standard solution (IS, 1 μg/mL) and 10 μL enzymatic hydrolysis cocktail (β‐glucuronidase/sulfatase mixture: 200 U/2 U enzymatic activity). The mixture underwent vortex mixing followed by 1‐h enzymatic digestion at physiological temperature (37°C). Subsequent protein precipitation was achieved through methanol addition (300 μL) with vigorous shaking. After phase separation by centrifugation (5000 rpm, 5 min), the organic supernatant was carefully decanted and subjected to nitrogen‐stream evaporation until completely dry. The residue was then reconstituted in 100 μL of mobile phase consisting of aqueous 0.1% FA‐ACN (v:v = 95:5) using ultrasonic‐assisted dissolution. Prior to UPLC‐MS/MS analysis, the final solution underwent high‐speed clarification centrifugation (13,000 rpm, 10 min) to remove particulate matter. The drug concentration‐time profiles were constructed, and pharmacokinetic parameters were analyzed using DAS 3.2.8 software.

### 2.8 Cell Culture, Transfection, and Treatments

The experiment used the human neuroblastoma SH‐SY5Y cell line to construct a stable transfected cell line. Cells were grown in a 1:1 MEM/F12 medium blend with added 15% fetal bovine serum for nutritional support. To avoid bacterial contamination, 500 IU/mL of penicillin and 500 μg/mL of streptomycin were incorporated. Cells were cultured at 37°C with 5% CO_2_ to maintain a pH of 7.2–7.4. The cell passage ratio was 1:2 to 1:4, and medium was replaced every 48–72 h to maintain the cells in the logarithmic growth phase. Routine testing confirmed that the cells were free from mycoplasma, bacterial, and fungal contamination. For cell freezing, a cryopreservation solution consisting of 60% base medium, 30% FBS, and 10% DMSO was used, and cells were stored in liquid nitrogen for long‐term preservation. The human Nrf2 knockdown plasmid, crafted by GeneChem, was used to transfect SH‐SY5Y cells seeded at a concentration of 100,000 cells per milliliter, utilizing the optimal amount of Lipofectamine 3000 (Invitrogen) in accordance with the protocol. 48 h subsequent to transfection, cells were exposed to 1 mM MPP^+^ in the culture environment, and subsequently treated with 4 μg/mL BYQZF for another 48 h.

### 2.9 Real‐Time qRT‐PCR

Cell samples and mouse brain tissue samples were immersed in TRIzol reagent for RNA extraction. RNA purity was measured using a spectrophotometer, ensuring that the absorbance ratio at 260 nm to 280 nm fell within the range of 1.8 to 2.0. cDNA generation was performed using a reverse transcription kit, and appropriate primers were used for amplification. Three technical replicates were performed for each sample, and a no‐template control was included to exclude contamination. The primer sequences (Table 3) for *Keap1*, *Nrf2*, and *GAPDH* were designed and validated by the company for specificity, and GAPDH acted as the internal control. The amplification program included pre‐denaturation, denaturation, annealing, and extension stages to obtain the *C*t values for the target genes. Gene expression was quantified relative to the control group and normalized accordingly. Each experiment was conducted a minimum of three times.

**TABLE 3:** Sequences of primers used for qRT‐PCR.

Name	Sequences
h*Nrf2*‐F	ATGACAATGAGGTTTCTTCGGC
h*Nrf2*‐R	CTTGCTCAATGTCCTGTTGCAT
h*GAPDH*‐F	CTTTGTCAAGCTCATTTCCTGG
h*GAPDH*‐R	TCTTCCTCTTGTGCTCTTGC
m*Nrf2*‐F	CGCCCTCAGCATGATGGACTTG
m*Nrf2*‐R	ACTTGTACCGCCTCGTCTGGAC
m*Keap1*‐F	AAGCACTTGGAATACCTGAGCACTG
m*Keap1*‐R	GATGACTTCTTCTGCCGCCTCTTC
m*GAPDH*‐F	AGAAGGTGGTGAAGCAGGCATC
m*GAPDH*‐R	CGAAGGTGGAAGAGTGGGAGTTG

### 2.10 Immunofluorescence Analysis

Brain tissue was fixed in 10% formaldehyde in PBS at 4°C for 24 h, dehydrated in gradient sucrose solutions (10%, 20%, and 30%), embedded, and sectioned into 6 μm frozen slices. Antigen retrieval was performed using citrate buffer (pH 6.0) at 95°C for 10 min. Sections were washed with PBS and incubated overnight at 5°C with primary antibody (1:1000 in PBS with 1% BSA). After washing, sections were incubated with fluorescence‐labeled IgG secondary antibody (1:1000) for 60 min. Nuclei were stained with DAPI, and sections were mounted with mounting medium. Images of the SN and striatum were captured using a confocal microscope. ImageJ software was used to quantify the number and average density for comparison among groups.

### 2.11 TUNEL

10% formaldehyde was used to fix protein and cell structure in mouse brain tissue (4°C for 24 h), followed by dehydration in increasing concentrations of alcohol solutions. The tissue was embedded in paraffin and sectioned into slices with a thickness of 6 μm along the coronal plane for subsequent staining. After fixation and washing with PBS (10 min, 3 times) and permeabilization (4°C, 5 min), TUNEL staining was performed using a commercially available TUNEL kit. Sections were incubated at 37°C for 60 min. After staining, rinse the tissue sections three times for 5 min each with PBS and incubate the sections in a controlled incubator at 37°C for 1 h. Repeat the washing and incubation steps. Use a fluorescence microscope to observe the sections, where TUNEL‐positive cells will exhibit green fluorescence, indicating cell apoptosis. Randomly select fields in the SN region to count the number of TUNEL‐positive cells, and calculate the apoptosis rate for each field. All image analysis is conducted using ImageJ software, and the average value from multiple sections is taken as the apoptosis data for each animal, which is further used for intergroup comparison and statistical analysis.

### 2.12 Western Blotting

Tissue samples underwent lysis in RIPA buffer containing protease inhibitors. To eliminate insoluble debris, the homogenates were centrifuged at 12,000 × g for 15 min under cold conditions (4°C). Protein concentration was determined using the BCA assay by measuring the absorbance of the purple complex at 562 nm and calculated using a standard curve. Protein samples were mixed with SDS sample buffer in a 1:1 ratio and denatured at 95°C for 5 min. After SDS‐PAGE separation, proteins were immobilized onto PVDF membranes, which were then incubated for an hour in 5% skim milk at ambient temperature to avoid non‐specific attachment. The membranes were incubated overnight with specific primary antibodies at 4°C. On the subsequent day, TBST was used to wash the membranes at ambient temperature and treated using a goat anti‐rabbit secondary antibody conjugated with HRP (1:2000) for 2 h at ambient temperature, away from light. Signals were detected using ECL (Enhanced chemiluminescence) Plus reagent and captured with a gel imaging system. ImageJ software was used to analyze the protein density, with GAPDH or β‐actin as references. Three repetitions of the experiment were performed. The uncropped, original scans of all Western blots are provided in Figure S5.

### 2.13 Co‐Immunoprecipitation

In this experiment, to investigate the protein interaction between Nrf2 and Keap1, co‐immunoprecipitation was used. The treated tissues or cells were cleansed with PBS to clear away any residual culture medium and then placed on ice and lysed with immunoprecipitation‐specific lysis buffer for 20–30 min to fully release the proteins. The lysates were subjected to high‐speed centrifugation, and the supernatant was collected. BCA reagent was incubated with the tissue or cell samples at 37°C for 40 min to measure the absorbance of the complex and generate a standard curve to determine protein concentration, adjusting to the required concentration. Under low‐temperature conditions (4°C), equal amounts of total protein were incubated with Keap1 antibody and rotated overnight to facilitate antigen–antibody binding. The next day, the mixture was incubated with pre‐equilibrated agarose beads (which had been pre‐coated with protein A/G and blocked with BSA) at the same temperature to capture the immune complexes. After the reaction, the immunoprecipitation system was washed multiple times to remove nonspecific binding components. Finally, the bound proteins were eluted using lysis buffer or sample loading buffer, and the eluates were analyzed by Western blot.

### 2.14 Elisa

Under 37°C conditions, samples were diluted 1:1000 and incubated with standards for 1 h. Three PBS washes were performed on the samples to get rid of unbound components. Afterward, HRP‐conjugated secondary antibodies were added, and incubation continued at 37°C for 1 h. The samples were mixed with a colorimetric substrate and incubated in the dark at 25°C for 20 min. After incubation, the absorbance values were read. A standard curve was used to calculate the specific concentrations of 8‐OHdG, GSSG, GSH, and CAT.

### 2.15 Statistical Methods

Statistical analyses were performed using R (4.2.1 version). Normality and homoscedasticity were assessed using the Shapiro–Wilk test and Brown‐Forsythe test, respectively. For comparisons between two groups of normally distributed data, the independent Student's *t*‐test was used. For non‐normally distributed data, the Wilcoxon Rank Sum Test (Mann–Whitney U Test) was applied. For comparisons among three or more groups, one‐way ANOVA followed by Tukey's post hoc test was used for normally distributed data. For non‐normally distributed data, the Kruskal‐Wallis test followed by Dunn's post hoc test was applied. *p* values from post hoc multiple comparisons were adjusted using the two‐stage step‐up method of Benjamini, Krieger, and Yekutieli to control the false discovery rate (FDR). Correlation analysis was performed using Spearman's rank correlation coefficient. A two‐sided *p*‐value of less than 0.05 was considered statistically significant, unless otherwise stated.

## 3 Results

### 3.1 Toxicity and Safety Assessment Results of BYQZF

The morphology and structure of the vital organs (heart, liver, spleen, lung, kidney, stomach, intestines, and brain) in different groups were observed by H&E staining. The morphology and structure of the vital organs (heart, liver, spleen, lung, kidney, stomach, intestines, and brain) of the control group were intact, the cell morphology was normal, the cytoplasm was evenly distributed, and the nucleus was regular and round. However, in MPTP‐intervened groups, the cell arrangement was loose, and the cytoplasmic content was reduced to some extent. Compared with the control group, the morphology and structure of the vital organs in the low and high concentration BYQZF treatment groups were not significantly different, indicating BYQZF was not toxic to vital organs (Figure S1).

Blood samples were collected from all mice, and the levels of ALT, AST, CREA, LDH, CK, IL‐6, TNF‐α, and IL‐1β were assessed. (Figure S2). The results showed that the levels of ALT, AST, LDH, and CK were similar to those in the control group in the low‐ and high‐ concentration BYQZF treatment groups without MPTP, and significantly increased in the MPTP groups decreasing after Madopar and BYQZF treatment. The secretion of IL‐6, TNF‐α, and IL‐1β was significantly increased in MPTP groups and significantly decreased in the MPTP + Madopar and MPTP + BYQZF‐L treatment groups compared with MPTP groups. The above serum biochemical analysis and inflammatory factor detection results showed that BYQZF can not cause obvious liver function injury, renal function injury, cardiomyocyte injury, and inflammatory reaction.

### 3.2 Results of UPLC‐MS/MS

The chemical constituents of BYQZF were systematically characterized using UPLC‐MS/MS technology. A comprehensive literature review was conducted by consulting databases such as PubMed, ChemSpider, Chemical Book, and Web of Science to identify the chemical components of BYQZF. The chemical structures of the identified compounds were elucidated, and their corresponding information, including names, molecular formulas, and exact mass values, was systematically organized. This data formed the basis for constructing a specialized database of BYQZF's material composition. The typical mass spectrum chromatograms of BYQZF are shown in Figure S3. Based on the mass spectrometry analysis of the samples, in conjunction with comparisons against the self‐built database and corroborated by relevant literature, a total of 472 compounds were successfully identified in BYQZF (Table S1). These included 97 carbohydrates and glycosides, 59 phenylpropanoids, 57 amino acids, peptides and derivatives, 45 terpenes, 39 fatty acyls and others. The results exhibited good repeatability, confirming the component stability and quality controllability of the BYQZF preparation.

### 3.3 Pharmacokinetic Evaluation

Based on the above chemical composition study (Section 3.2), the prototypical components of BYQZF in the normal mice and PD model mice were studied. A UPLC‐MS/MS quantitative analysis method was used to study the pharmacokinetics of 10 major components in plasma samples of mice after oral administration of BYQZF at a single dose of 5 g crude drug/kg. The results showed that there were significant differences in the pharmacokinetic parameters of *C*_max_, AUC_(0–t)_ and MRT_(0‐t)_ of some components in the BYQZF treatment without MPTP group and the MPTP + BYQZF treatment group (Table S2). Comparison of AUC_(0‐24h)_ values revealed significantly enhanced systemic absorption for most compounds in the BYQZF treatment without MPTP group compared to the MPTP + BYQZF treatment group (Figure S4). The results showed that compared with the BYQZF treatment without MPTP group, the pharmacokinetics of 8‐epiloganic acid, 3‐hydroxyflavone, hyperin, manninotriose, L‐glutamic acid, inositol, uracil, mangiferol, isoacteoside and L‐β‐aspartyl‐L‐aspartic acid in the MPTP + BYQZF treatment group were significantly different (*p* < 0.05), suggesting that the pathological conditions of PD significantly affected the pharmacokinetic behavior of BYQZF in vivo, providing experimental data support for clinical medication.

### 3.4 BYQZF Alleviates Motor Symptoms in MPTP‐Induced PD Mice

**FIGURE 1:**
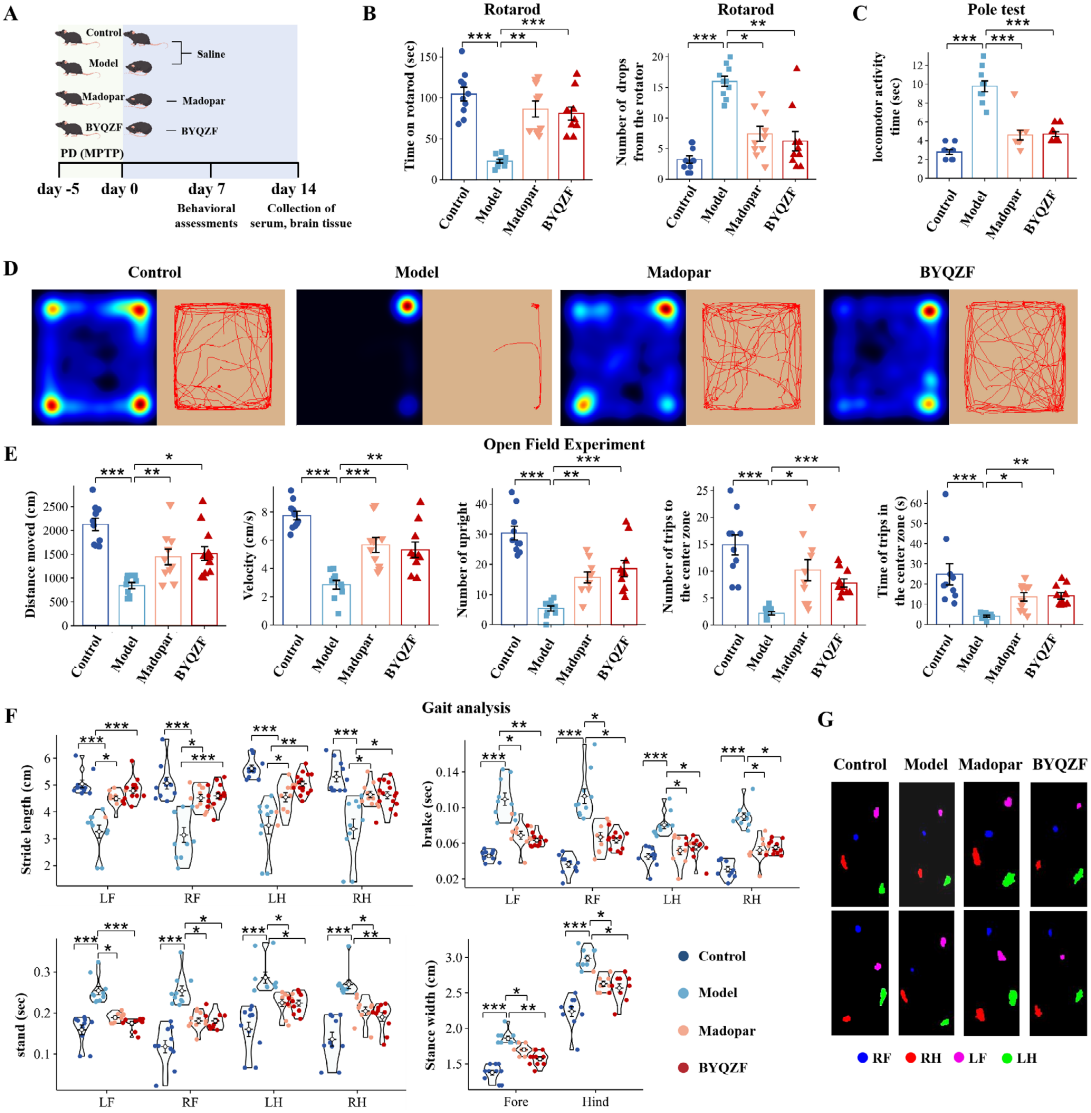
BYQZF alleviates motor symptoms in MPTP‐induced PD mice. (A) Diagram showing the experimental procedure for preventing MPTP‐induced PD with BYQZF. (B) Time on rotarod (sec) and number of drops from the rotator in 5 min. (C) Time of pole test (sec). (D) Heatmap and movement trajectory plot of the open field test. (E) Statistical analysis of distance moved, velocity, the number of upright, the number, and the time of trips to the center zone. (F, G) Gait analysis. *n* = 10 in each group. LF, left fore limb. LH, left hind limb. RF, right fore limb. RH, right hind limb. *p < 0.05, **p < 0.01, ***p < 0.001.

To verify the effect of BYQZF on PD, in vivo experiments in MPTP‐induced PD mice were conducted. Figure 1A–C showed the outcomes of the rotarod test and pole tests. In the rotarod test, the model group mice exhibited a notable decrease compared to the control group in retention time on the rotating rod (*p* < 0.001), indicating a marked decline in motor coordination. Treatment with Madopar and BYQZF significantly prolonged retention time. Meanwhile, the model group mice exhibited significantly more falls during the test as opposed to the control group (*p* < 0.001), while the administration of BYQZF led to a notable decrease in the number of falls in comparison to the model group (*p* < 0.01), indicating an improvement in motor stability. In the pole test, when contrasted with the control group, the model group mice required markedly more time to descend (*p* < 0.001) and reflected clear motor slowing. Both Madopar and BYQZF treatments significantly reduced the time to descend (*p* < 0.001); there was no notable difference between the two treatments. Figure 1D,E showed the data from the open field test. The heatmap and movement trajectory plot from the open field experiment displayed that the model group mice had significantly reduced activity range and exploratory behavior. In contrast, both Madopar and BYQZF‐treated mice exhibited increased activity, and their movement paths closely resembled those of the control group, suggesting improvements in spontaneous movement and spatial exploration ability. Quantitative analysis of the open field test showed a significant decrease in total distance traveled, speed, rearing frequency, as well as the number and duration of entries into the center zone in the model group mice relative to the control group (*p* < 0.001). BYQZF treatment markedly increased the mice's total distance traveled (*p* < 0.05), speed (*p* < 0.01), and rearing frequency (*p* < 0.01). Additionally, BYQZF markedly increased the frequency of entries into the center zone (*p* < 0.001) and the duration in the center (*p* < 0.01), indicating its potential to alleviate motor inhibition and anxiety‐like behaviors. The gait analysis results demonstrated that after treatment with BYQZF, the stride length significantly increased, whereas brake, stand, and stance width significantly decreased (Figure 1F,G). These results showed that BYQZF significantly alleviated motor symptoms in PD mice.

### 3.5 BYQZF Ameliorated MPTP‐Induced DA Neuronal Impairment in the Nigrostriatal Path

L‐tyrosine is converted into L‐3,4‐dihydroxyphenylalanine, a dopamine precursor, by TH. Dopamine synthesis and release are impacted by the loss or reduced expression of TH. Inability to generate dopamine or lack sufficient dopamine secretion may result from PD. The results in Figure 1A,B showed that the striatum and SN of control mice had abundant TH‐positive neurons, with strong fluorescence signals and intact morphological structures. In contrast, the model group revealed considerably reduced TH fluorescence signals, indicating substantial decline in dopaminergic neurons and neurodegenerative damage caused by MPTP treatment. As depicted in Figure 1C,D, the model group showed a considerable reduction in the amount of TH‐positive cells within the striatum and SN relative to the control group (*p* < 0.001 and *p* < 0.01). In comparison to the model group, the amount of TH‐positive cells was significantly restored after intervention with Madopar and BYQZF (*p* < 0.001). Western blot was used to detect the change trend of TH protein expression in the midbrain of mice in different groups. The results showed a trend similar to that observed in TH (+) immunofluorescence staining. These results suggest that BYQZF significantly improves DA neuronal damage in PD.

### 3.6 BYQZF ameliorated MPTP‐induced α‐synuclein aggregation, neuroinflammation, apoptosis and mitochondrial dysfunction

**FIGURE 2:**
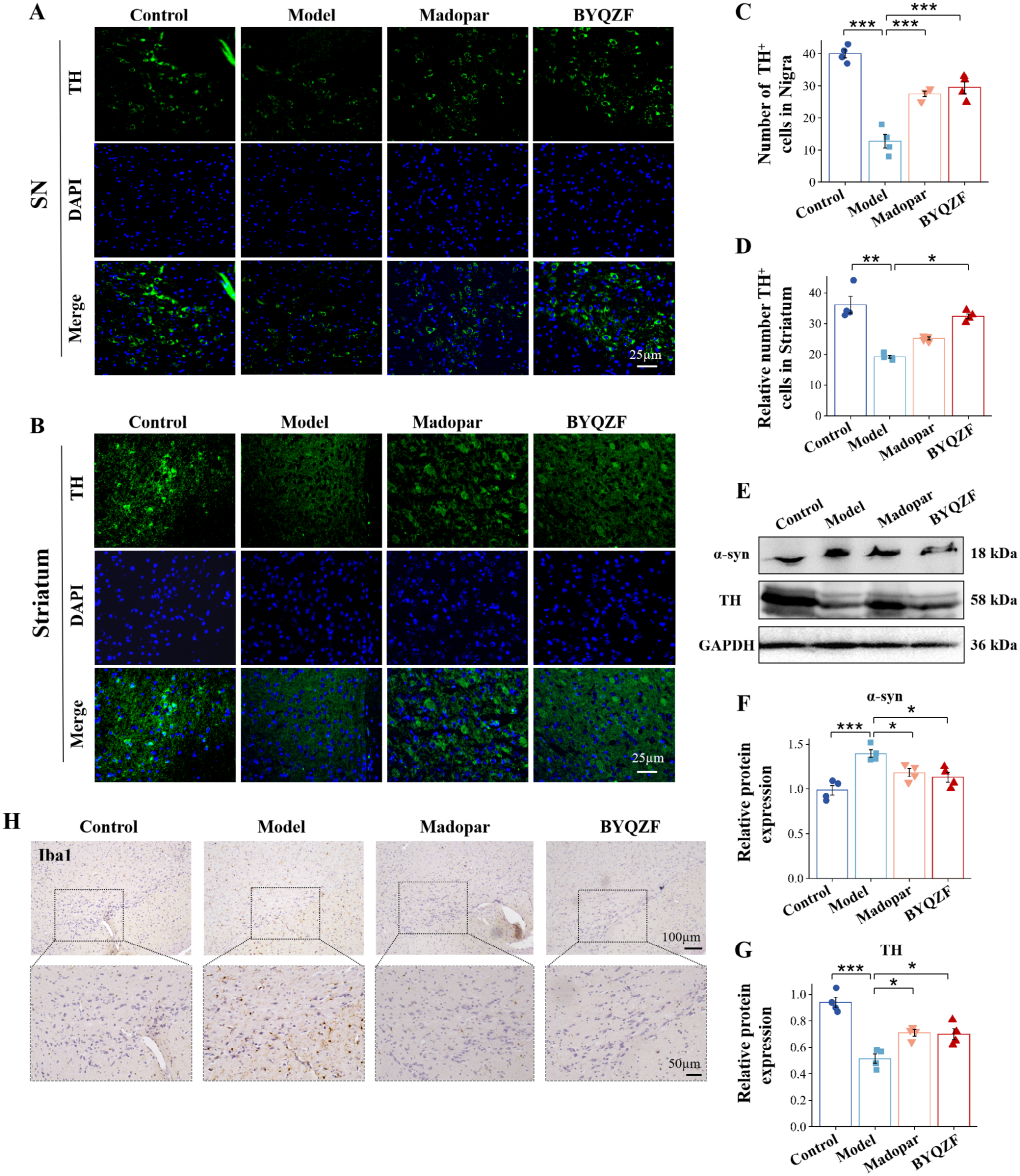
Effects of BYQZF on DA neurons in the SN and striatum of PD mice models. (A, B) Immunofluorescence staining of DA neurons in the SN and striatum of the mice. (C, D) Statistics of DA neurons. (E–G) Expression levels of TH and α‐syn in the midbrain by Western blot analysis. (H) Immunohistochemistry staining of Iba1^+^cells in the midbrain of the mice. *n* = 3–6 for each group. The data were expressed as mean ± SEM. *p < 0.05, **p < 0.01, ***p < 0.001.

Western blot was used to detect the expression of α‐syn protein in the midbrain of mice in different groups (Figure 2E,F). The results showed that compared with the control group, the expression of α‐syn protein in the MPTP group was significantly increased, and the expression of α‐syn protein was significantly decreased after treatment with BYQZF and Madopar. These results indicate that BYQZF can effectively alleviate α‐syn protein aggregation. The morphology of microglia in the SN was observed by immunohistochemical staining (Figure 2H). The results showed that compared with the control group, the soma of microglia in the SN of the MPTP group was larger, and the cells changed from branched to “reactive” or amebic‐like morphology. After treatment with BYQZF and Madopar, the activation state of microglia was suppressed. At the same time, the expression of TNF‐α, IL‐6, and IL‐1β was detected by ELISA (Figure S2F,G). The results showed that compared with the control group, the inflammatory factors in the MPTP group were significantly increased (*p* < 0.001), and the inflammatory factors were significantly decreased after treatment with BYQZF (*p* < 0.001, *p* < 0.01, *p* < 0.05). The above results indicated that BYQZF could alleviate the neuroinflammation induced by MPTP.

**FIGURE 3:**
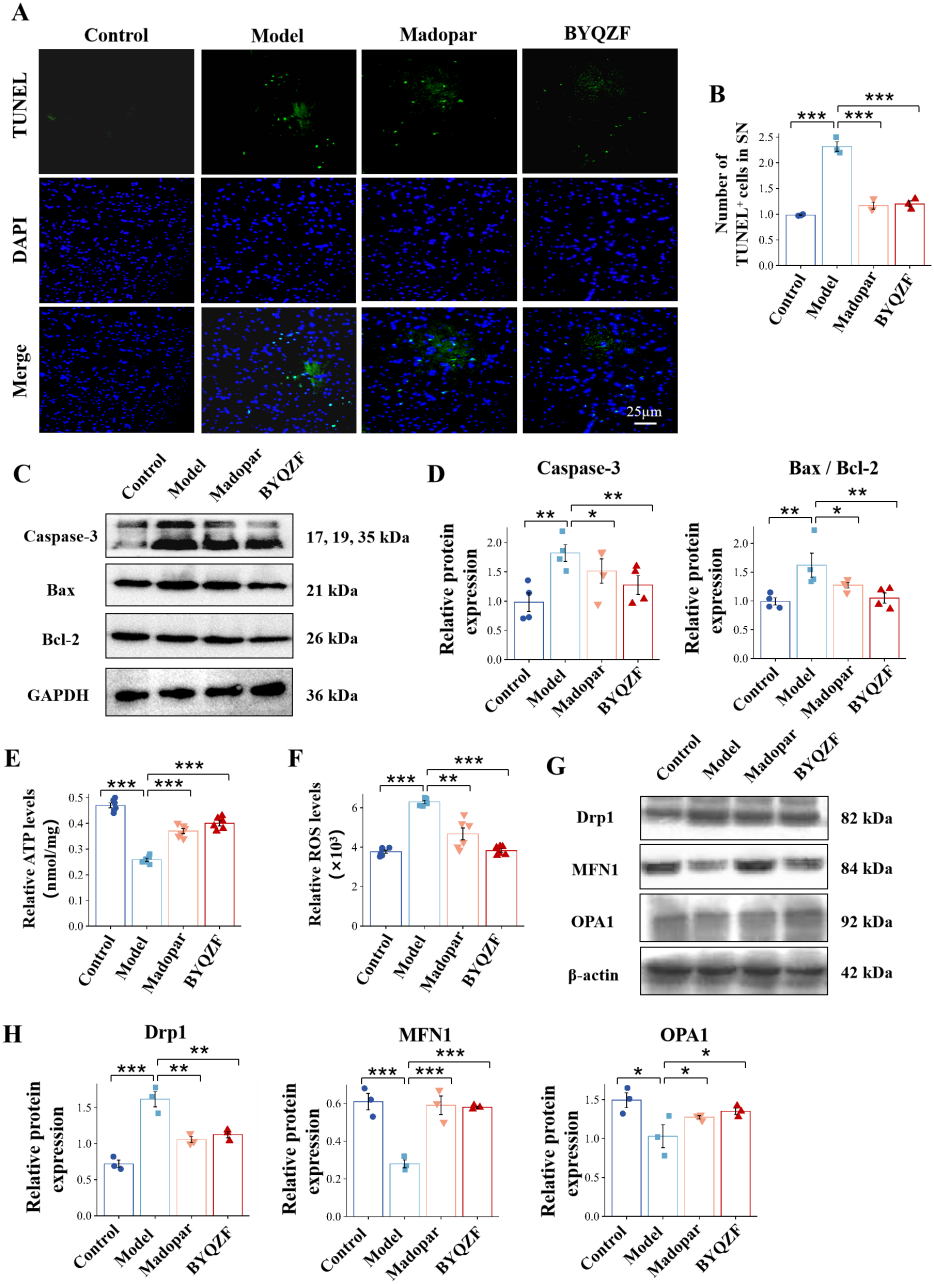
BYQZF ameliorated MPTP‐induced apoptosis and mitochondrial dysfunction of SN neurons. (A) Detection of apoptosis in SN neurons using TUNEL labeling (scale bar: 25 μm). (B) Relative number of TUNEL (+) cells in the SN. (C, D) Expression levels of Bcl‐2, Caspase‐3, and Bax in the midbrain by Western blot analysis. (E) Relative ATP levels (nmol/mg) in the midbrain. (F) Relative ROS levels (×10^3^) in the midbrain. (G, H) Expression levels of Drp1, MFN1, and OPA1 in the midbrain by Western blot analysis. *n* = 3–6. The data are presented as mean ± SEM. *p < 0.05, **p < 0.01, ***p < 0.001.

Apoptosis of SN neurons was assessed by TUNEL labeling assays. TUNEL fluorescence labeling (Figure 3A,B) showed a significant increase in apoptotic cells (green signals) in the midbrain SN of mice in the model group, while the number of apoptotic cells was notably reduced in the Madopar and BYQZF groups, particularly in the BYQZF group, indicating that BYQZF effectively inhibits apoptosis. The results of Western blot revealed that caspase‐3 and bax expression was notably elevated in the model group, while the expression of bcl‐2 showed the opposite trend, indicating a clear activation of apoptosis in the model mice. In the Madopar and BYQZF groups, the expression levels of caspase‐3 and the Bax/Bcl‐2 ratio were less than those in the model group (*p* < 0.01), further demonstrating the potential of BYQZF in inhibiting neuronal apoptosis.

The relative ATP levels of midbrain tissues in mice were detected (Figure 3E). The results showed that compared with the control group, the level of mitochondrial ATP in the MPTP group was significantly decreased (*p* < 0.001); compared with the MPTP group, the mitochondrial ATP levels were significantly increased in the BYQZF group and the Madopar group (*p* < 0.001). The expression trend of relative ROS level was opposite to the relative ATP levels (Figure 3F). We detected the expression of mitochondrial‐associated fission proteins and fusion proteins (Figure 3G,H), and the results showed that compared with the control group, the expression of Drp1 protein was significantly increased, and the expression of MFN1 and OPA1 protein was significantly decreased in the MPTP group (*p* < 0.001, *p* < 0.001, *p* < 0.05). Compared with the MPTP group, Drp1 protein expression was significantly decreased in the BYQZF group and Madopar group (*p* < 0.01), while MFN1 and OPA1 protein expression was significantly increased (*p* < 0.001, *p* < 0.05). The above results showed that BYQZF treatment could ameliorate the mitochondrial dysfunction of PD mice.

### 3.7 BYQZF Treatment Diminished the Oxidative Stress‐Related Damage and Promoted the Downstream Antioxidant Enzyme Protein Expressions

**FIGURE 4:**
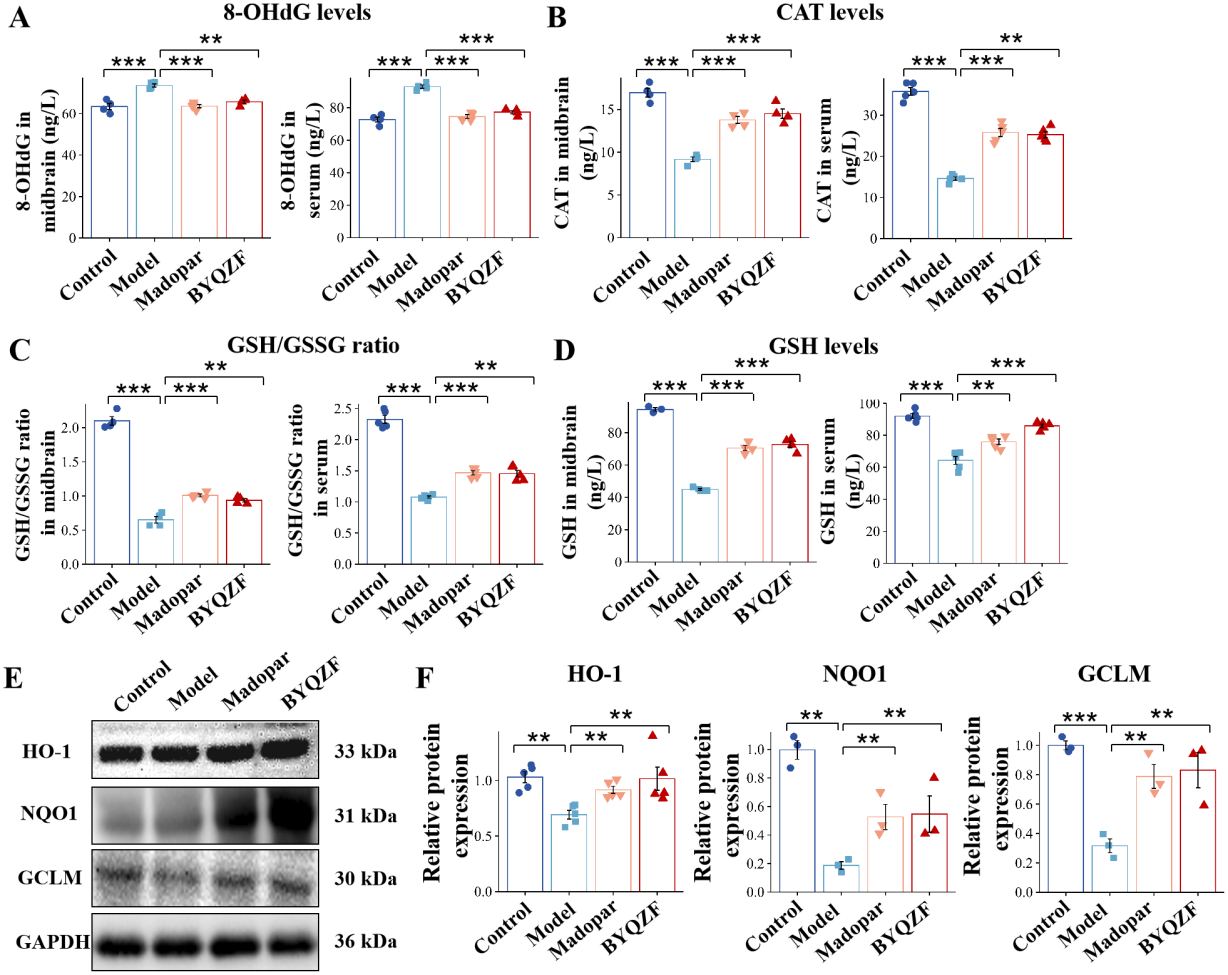
BYQZF treatment alleviated the oxidative stress damage and promoted the downstream antioxidant enzyme protein expressions. 8‐OHdG (A), CAT (B), GSH/GSSG ratio (C), GSH (D) levels in the midbrain and serum. (E) Western blotting analysis of HO‐1, NQO1, and GCLM. (F) Relative expression levels of HO‐1, NQO1, and GCLM proteins. *p < 0.05, **p < 0.01, ***p < 0.001.

The serum and midbrain of all mice were gathered and ELISA was conducted for 8‐hydroxy‐2′‐deoxyguanosine (8‐OHdG), Catalase (CAT), GSH and GSSG (Figure 4A–D). 8‐OHdG serves as a crucial indicator for evaluating oxidative damage to DNA. Compared to the control group, the 8‐OHdG levels in the model group were notably raised (*p* < 0.001), suggesting that MPTP treatment induced significant DNA oxidative damage. After intervention with Madopar and BYQZF, the 8‐OHdG levels decreased significantly and returned to levels close to the control group. CAT, a key antioxidant enzyme that removes hydrogen peroxide, reflects the body's antioxidant capacity. The CAT levels in the model group were significantly reduced (*p* < 0.001), suggesting that the antioxidant enzyme system was suppressed following MPTP treatment. After treatment with BYQZF, CAT levels were significantly improved (*p* < 0.01). The comparison of reduced glutathione to its oxidized form (GSH/GSSG) is a classic indicator of cellular redox status. The GSH/GSSG ratio in the model group was markedly reduced (*p* < 0.001). BYQZF intervention significantly increased the GSH/GSSG ratio in both the midbrain and serum (*p* < 0.01). GSH is one of the major non‐enzyme antioxidants within cells and directly participates in scavenging ROS. The absolute GSH levels in the model group were substantially less, indicating a significant redox imbalance. In the midbrain, BYQZF significantly increased the GSH levels in the serum (*p* < 0.001). The above results suggest that BYQZF has a good antioxidant capability, effectively inhibiting oxidative stress damage and protecting neurons.

To investigate the molecular mechanism of BYQZF's antioxidant effect further, we assessed the expression of Nrf2 signaling pathway‐related downstream proteins—HO‐1, NQO1, and GCLM (Figure 4E,F). HO‐1 degrades ROS, NQO1 prevents ROS formation by acting as an NAD(P)H dehydrogenase, and GCLM acts as a regulatory component of the enzyme that limits the rate of glutathione synthesis. Western blot results revealed that, in comparison to the control group, the model group had suppressed GCLM expression. GCLM, one of the key enzymes for glutathione synthesis, is typically upregulated via the Nrf2 pathway under oxidative stress to boost the cell's antioxidant capacity. The inhibition of GCLM expression in the model group may reflect a weakened or suppressed Nrf2 pathway, thus impairing the cell's antioxidant defense system. In contrast, BYQZF significantly increased the levels of HO‐1 and NQO1 proteins, suggesting that BYQZF may initiate the Nrf2/ARE pathway to increase the expression of enzymes that act as antioxidants downstream, thereby exerting its antioxidant and neuroprotective effects. In terms of relative protein expression, compared to the control group, the amount of expression of HO‐1, NQO1, and GCLM in the model group was considerably reduced (*p* < 0.01). After treatment with BYQZF, the expression levels of HO‐1, NQO1, and GCLM improved significantly (*p* < 0.01). These results showed that BYQZF treatment significantly alleviated the oxidative stress damage and promoted the downstream antioxidant enzyme protein expressions.

### 3.8 BYQZF Treatment Promoted the Dissociation of Keap1/Nrf2 and the Nuclear Translocation of Nrf2

**FIGURE 5:**
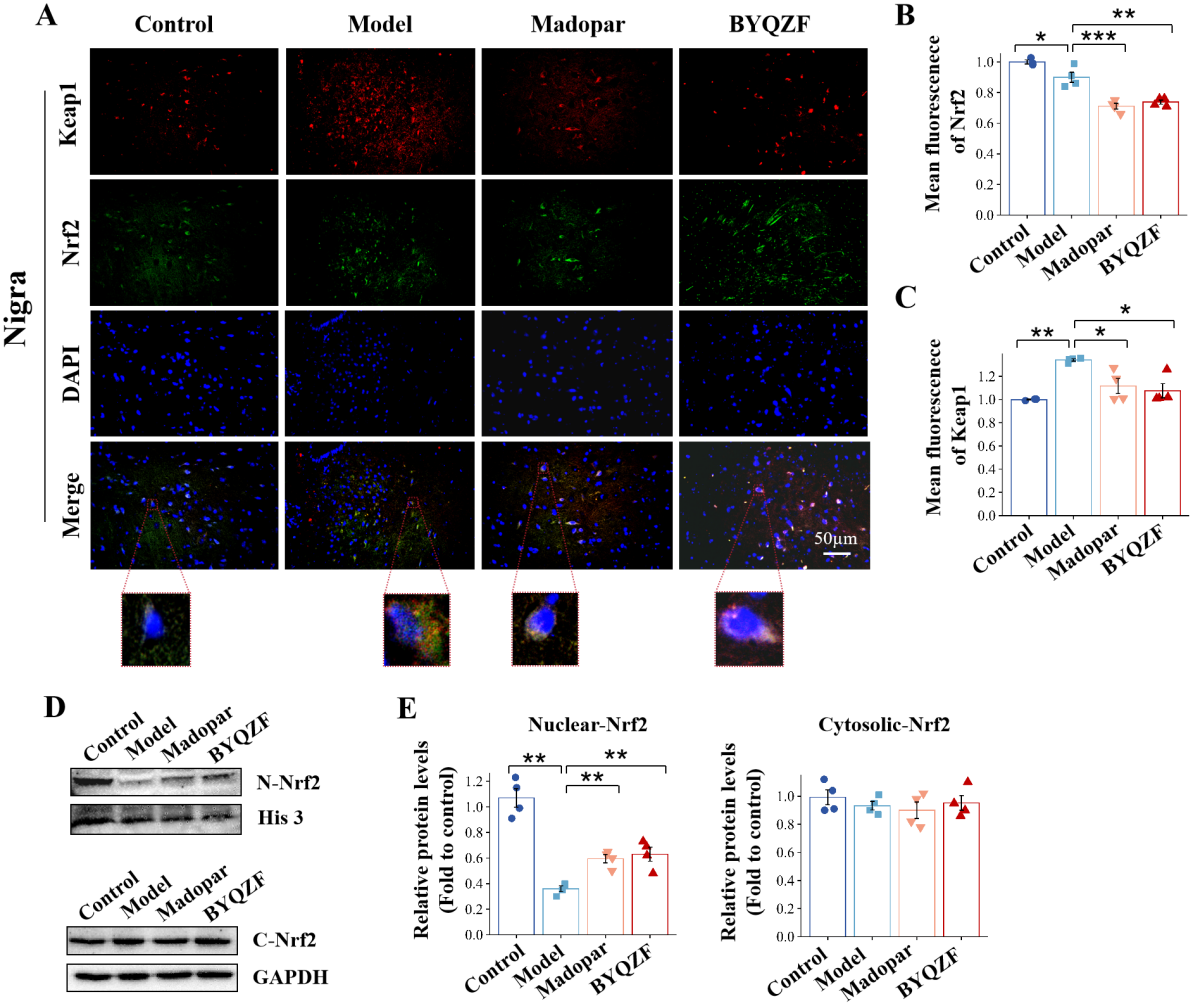
BYQZF treatment promoted the nuclear translocation of Nrf2. (A) The immunofluorescence results of Nrf2 and Keap1 proteins in SN. (B, C) Statistical results of the mean fluorescence intensity of the immunofluorescence. (D) The western blot results of nuclear‐Nrf2 and cytosolic‐Nrf2 proteins in SN. (E) Statistical results of the relative intensity of the WB. *p < 0.05, **p < 0.01, ***p < 0.001.

**FIGURE 6:**
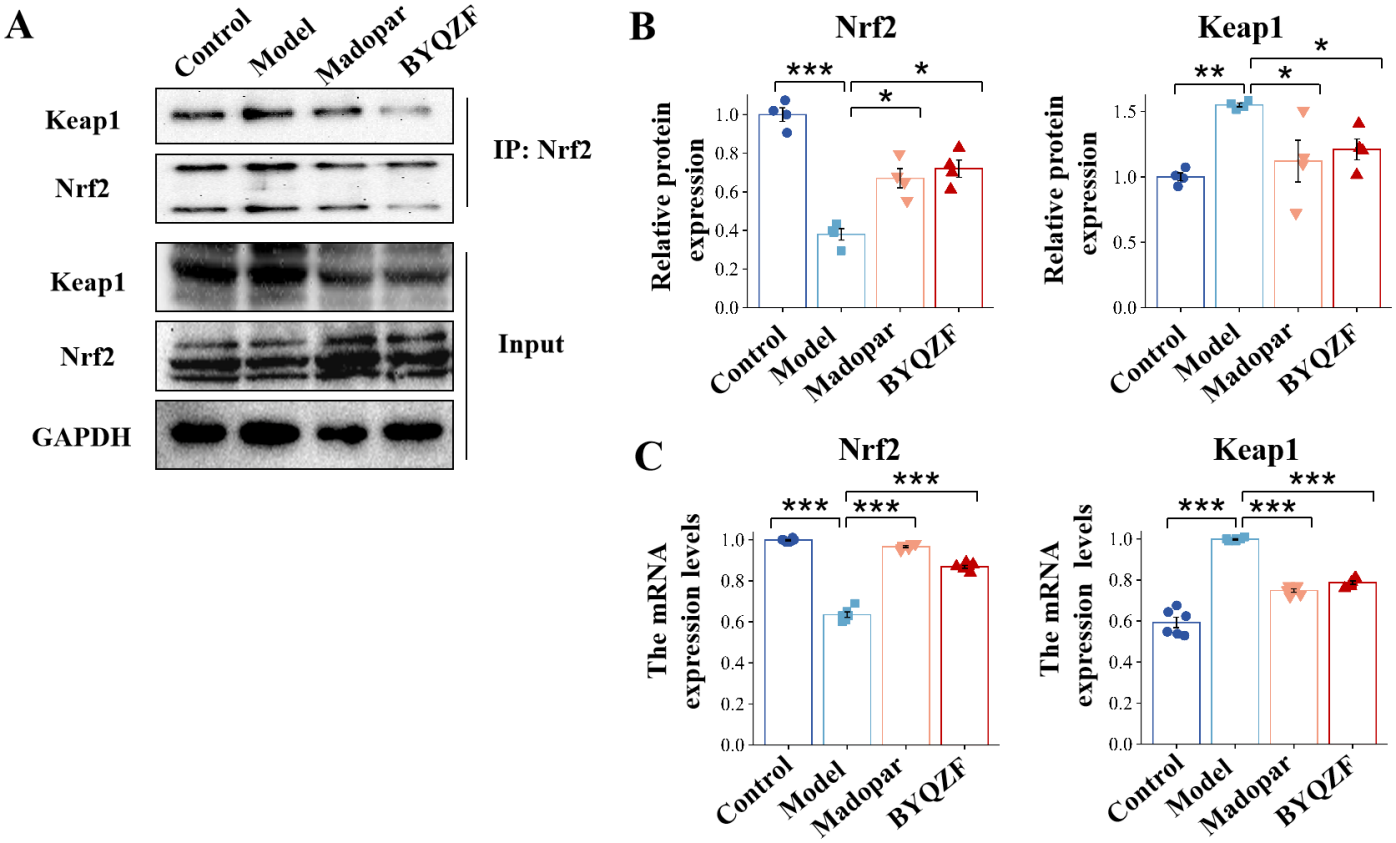
BYQZF treatment promoted the dissociation of Keap1/Nrf2. (A) Co‐IP assay results. (B) Statistical results of the relative intensity of the WB. (C) the RT‐qPCR analysis results of *Nrf2* and *Keap1* mRNA. *n* = 4–6. The values are shown as the means ± SD. *p < 0.05, **p < 0.01, ***p < 0.001.

The typical activation process of Nrf2 involves the transfer from the cytoplasm into the nucleus. Therefore, this study examined Nrf2's transfer to the nucleus in SN using immunofluorescence staining and western blot. Immunofluorescence staining was used to assess the relative expression of Nrf2 and Keap1 in the SN (Figure 5A–C). The staining outcomes revealed that, compared to the model group, the expression levels of Nrf2 in the BYQZF group were markedly enhanced, while the expression of Keap1 showed the opposite trend. In the immunofluorescence staining images, green represents Nrf2, and blue represents the nucleus. The co‐localization results of Nrf2 with the nucleus indicated that Nrf2 is more expressed in the cytoplasm in the control group. After BYQZF intervention, the expression of Nrf2 in the nucleus increased, indicating BYQZF treatment facilitated the movement of Nrf2 into the nucleus. Western blot analysis was conducted to determine Nrf2 expression levels in the nucleus and cytoplasm (Figure 5D,E). After BYQZF treatment, the Nrf2 content in the nucleus was markedly higher than the model group (*p* < 0.01), indicating that BYQZF enhanced Nrf2 nuclear translocation and antioxidant function by facilitating the dissociation of the Keap1/Nrf2 complex. As shown in Figure 4E,F, HO‐1, NQO1 and GCLM, three classical subsequent factors in the Nrf2 pathway, were considerably elevated when treated with BYQZF, also demonstrating that BYQZF promoted the movement of Nrf2 into the nucleus, subsequently activating the Nrf2 pathway. Western blot analysis in Figure 6B,C revealed that, relative to the control group, Keap1's expression in the model group was notably elevated (*p* < 0.001), implying that under oxidative stress conditions, the upregulated Keap1 may inhibit Nrf2 activity and translocation by binding to Nrf2. Meanwhile, western blot analysis confirmed that Nrf2 expression levels in the cytoplasm did not significantly differ among the groups (*p* > 0.05). After BYQZF intervention, the expression of Keap1 was significantly decreased (*p* < 0.01), indicating that BYQZF facilitated the breakdown of the Keap1/Nrf2 complex and reduced the negative regulation of Keap1, thus promoting Nrf2 stability.

Given that our findings showed BYQZF's neuroprotective effects were linked to the Nrf2/HO‐1 signaling pathway, we proceeded to examine the Keap1/Nrf2 complex levels and the separation of Nrf2 from the Keap1/Nrf2 heterodimer in the SN. As shown in Figure 6A, co‐immunoprecipitation (Co‐IP) assay results showed that the level of the Keap1/Nrf2 complex in the BYQZF group was observably decreased compared with the Model group, indicating that BYQZF aided in the dissociation of Nrf2 from the Keap1/Nrf2 complex. Equivalent findings of immunofluorescence staining in Figure 5A showed that the model group exhibited a relatively low number and fluorescence intensity of Nrf2‐positive cells. These results suggested that under oxidative stress conditions, upregulated Keap1 may lead to Nrf2 binding and ubiquitination, inhibiting its activity. After BYQZF treatment, Keap1 expression significantly decreased, while Nrf2 expression was markedly upregulated, indicating that BYQZF promotes the dissociation of the Keap1/Nrf2 complex by reducing Keap1 expression, thus enhancing Nrf2 stability and activity. The levels of Nrf2 were significantly elevated in the BYQZF group in comparison to the model group (*p* < 0.05). This indicates that BYQZF promotes Nrf2 expression and may enhance Nrf2 activity by dissociating the Keap1/Nrf2 complex. In contrast, the levels of Keap1 in the BYQZF group were greatly lower than in the model group (*p* < 0.01), suggesting that BYQZF may promote Nrf2 activity by reducing Keap1 expression, thus improving the oxidative stress response. The mRNA levels of *Nrf2* and *Keap1* expression in the SN of mice are shown in Figure 6C. The *Nrf2* mRNA levels in the model group were considerably reduced relative to the control group (*p* < 0.001). The *Nrf2* mRNA levels in the BYQZF group were markedly higher than in the model group (*p* < 0.001), indicating that BYQZF enhanced Nrf2 function not only by upregulating its protein expression but also by promoting its transcription. In the model group, *Keap1* mRNA levels were notably elevated compared to the control group (*p* < 0.05), while the *Keap1* mRNA levels in the BYQZF‐treated group were lower than those in the model group (*p* < 0.01), suggesting that BYQZF may further dissociate the Keap1/Nrf2 complex by downregulating Keap1 transcription, thereby enhancing Nrf2 activity. These results showed that BYQZF treatment encouraged the separation of Keap1 and Nrf2 and the nuclear translocation of Nrf2.

### 3.9 Knockdown of Nrf2 Blocks the Neuroprotective Roles of BYQZF in MPP ^+^‐Induced PD Cell Models

**FIGURE 7:**
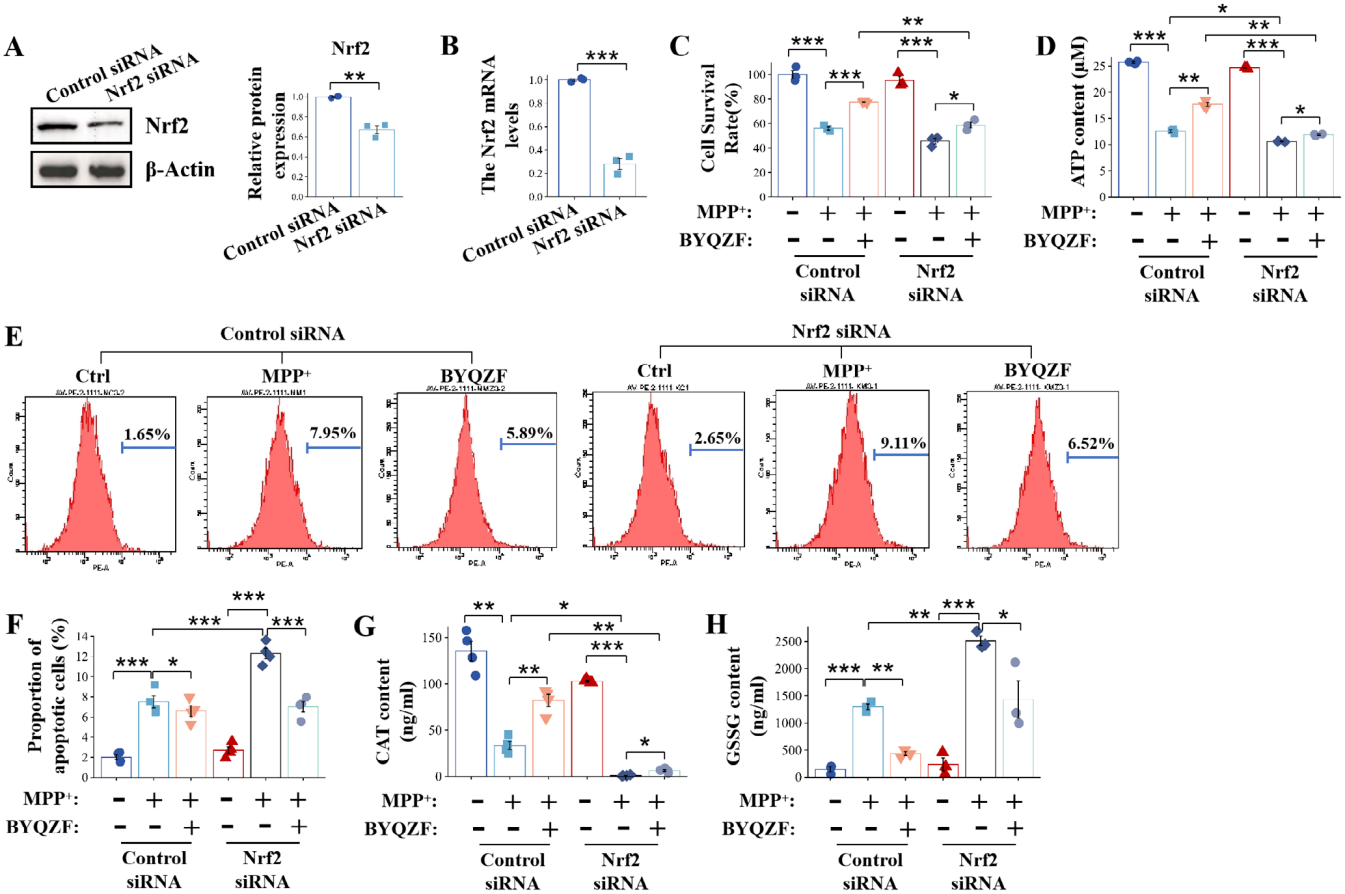
Knockdown of Nrf2 blocks the neuroprotective effects of BYQZF in MPP^+^‐induced PD cell models. (A, B) Nrf2 knockdown validated by Western blot and RT‐qPCR. (C) Cell survival rate. (D) ATP expression levels. (E, F) Flow cytometry to detect apoptosis rate. (G) CAT expression levels. (H) GSSG expression levels. *p < 0.05, **p < 0.01, ***p < 0.001.

As our results revealed that the neuroprotective impacts of BYQZF were linked to the Nrf2/HO‐1 signaling mechanism in vivo, we then investigated the molecular process of BYQZF in vitro models. Furthermore, we assessed the anti‐oxidative effects of BYQZF against MPP^+^ insults through Nrf2 knockdown via specific Nrf2 siRNA. Firstly, we validated the knockdown of Nrf2 by western blot and RT‐qPCR. Figure 7A,B showed that the Nrf2 protein and *Nrf2* mRNA levels were reduced after transfection with Nrf2‐siRNA of SH‐SY5Y cells compared with the control cells (empty vector transfection group). These results confirmed the effectiveness of the Nrf2 knockdown. Furthermore, we assessed cell death and apoptosis in the MPP^+^‐treated SH‐SY5Y cells and the impact of BYQZF intervention by CCK‐8 labeling (Figure 7C), ATP level assay (Figure 7D) and Annexin V‐PE apoptosis detection kit (Figure 7E,F). Figure 7C showed that MPP^+^ treatment markedly decreased the rate of cell survival (*p* < 0.05), which aligns with the oxidative stress and neuronal damage induced by MPTP. After treatment with BYQZF, cell survival was restored, and the oxidative stress induced by MPP^+^ was alleviated. Notably, under Nrf2 knockdown conditions (Nrf2 siRNA group), the restorative effect of BYQZF on cell survival was significantly weakened (*p* < 0.05), suggesting that the neuroprotective functions of BYQZF are contingent upon the activation of the Nrf2 signaling pathway. As a central regulator of the cellular oxidative stress response, the absence of Nrf2 significantly weakened BYQZF's protective effects. As shown in Figure 7D, MPP^+^ treatment led to a significant decrease in cellular ATP levels (*p* < 0.001), suggesting that MPP^+^ impairs mitochondrial function, suppressing cellular energy metabolism. Energy deficiency is a common phenomenon in PD models. After treatment with BYQZF, ATP levels markedly elevated (*p* < 0.01), but in the Nrf2 knockdown group, the restoration of ATP levels by BYQZF was significantly diminished (*p* < 0.01). This indicates that Nrf2 holds significant importance in maintaining cellular energy metabolism, and the absence of Nrf2 hinders BYQZF's ability to restore energy metabolism. Figure 7E,F showed that MPP^+^ treatment significantly increased the proportion of cells undergoing apoptosis (from 1.65% to 7.95%), indicating that MPP^+^ induces cell apoptosis through mitochondrial dysfunction and oxidative stress. After BYQZF intervention, the proportion of apoptotic cells decreased significantly (5.89%). Notably, after Nrf2 knockdown, the anti‐apoptotic effect of BYQZF was significantly weakened (6.52%), indicating that the Nrf2 signaling pathway is crucial in the anti‐apoptotic effects of BYQZF. As shown in Figure 7G, MPP^+^ treatment significantly reduced CAT activity (*p* < 0.01). After BYQZF intervention, CAT activity was significantly restored (*p* < 0.01), indicating that BYQZF alleviates oxidative stress by activating the antioxidant enzyme system. However, in the Nrf2 knockdown group, BYQZF's restoration of CAT activity was weakened (*p* < 0.01). GSSG is the oxidized product of glutathione in the antioxidant process and is commonly used as a biomarker of oxidative stress levels. Elevated GSSG levels typically indicate that the cell is in a high oxidative stress state and the redox balance is disrupted. Figure 7H showed that MPP^+^ treatment significantly increased GSSG levels (*p* < 0.001). After treatment with BYQZF, GSSG levels were considerably reduced (*p* < 0.01). In the Nrf2 knockdown group, BYQZF's effect on reducing GSSG levels was significantly weakened (*p* < 0.01). This indicates that Nrf2 is crucial for sustaining intracellular redox equilibrium as it modulates the expression of antioxidant enzymes and improves the GSH/GSSG ratio. These results indicate that knockdown of Nrf2 blocks the neuroprotective functions of BYQZF in MPP^+^‐induced PD cell models.

## 4 Discussion and Conclusion

Parkinson's disease (PD) ranks as the second most prevalent neurodegenerative disorder, following Alzheimer's disease [33]. The standardized incidence rate of PD is approximately 8–20 per 100,000 people, and it accounts for 3% of the total population in industrialized countries, with the highest incidence in high‐income North American countries [34]. With the advancement in age demographics, the prevalence of this condition is on the upward trend, posing a major challenge to the global public health system [34]. The etiology of PD is complex; the underlying mechanisms of its pathogenesis include the degeneration of dopaminergic neurons, the presence of oxidative stress, and mitochondrial dysfunction, among other factors [35]. Current treatments mainly rely on dopamine replacement therapy, which can temporarily alleviate symptoms but cannot delay disease progression. Clinical treatments still face limitations in efficacy, dependence, and significant side effects [36]. Therefore, exploring therapies with clear mechanisms, high safety, and long‐term intervention potential has become one of the key directions in PD research.

Bu‐Yin‐Qian‐Zheng Formula (BYQZF), a classic prescription for treating PD, has been shown in previous studies to improve oxidative stress [24, 25]. Nevertheless, the specific mechanism by which BYQZF confers neuroprotection and reduces oxidative stress against PD has not been clarified. In an effort to further elucidate the neuroprotective attributes of BYQZF and unravel its potential molecular mechanisms, our study was conducted. This study systematically investigates the mechanism of BYQZF's action on the Keap1/Nrf2/HO‐1 signaling pathway, enhances antioxidant defense, and protects neurons in MPTP‐induced PD mouse models and MPP^+^‐induced SH‐SY5Y PD cell models. It reveals the potential application of BYQZF as an adjunctive therapy in neurodegenerative diseases.

BYQZF is composed of *Rehmanniae radix praeparat*a, *Anemarrhenae rhizoma*, *Phellodendri chinensis* Cortex, *Testudinis plastrum*, *Typhonii rhizoma*, Scorpio, and *Bombyx* Batryticatus. Our previous HPLC analysis showed that BYQZF contains a variety of active components with antioxidant and neuroprotective effects, such as Berberine hydrochloride, Phellodendrine hydrochloride, Rehmannioside D, Acteoside, Mangiferin, and Timosaponin B [27]. Berberine hydrochloride, a typical isoquinoline alkaloid, can activate the Nrf2/HO‐1 pathway, improve hydrogen peroxide‐induced oxidative stress, scavenge intracellular ROS [37], and inhibit the expression of Cx43 in the spinal cord, alleviating vincristine‐induced neuroinflammation and mitochondrial damage [38]. After silencing Nrf2 or knocking out HO‐1, this effect was completely blocked, suggesting that this action is highly dependent on the Nrf2/HO‐1 pathway [37, 38]. Rehmannioside D, a characteristic component of Rehmannia, has been shown to inhibit the spontaneous oxidation of endogenous dopamine and further reduce oxidative byproducts generated during dopamine metabolism in the context of monoamine oxidase inhibition, thus exerting an antioxidant neuroprotective effect [39]. Acteoside, a phenolic acid compound, has been confirmed in PD models to activate the Nrf2/GPX4 pathway, significantly alleviating ferroptosis and lipid peroxidation, enhancing mitochondrial autophagy, and upregulating the expression levels of antioxidant enzymes such as GPX4 and SOD, thereby protecting neurons from damage induced by SAL (dopamine metabolic toxic products) [40, 41].

The components of BYQZF have been shown in previous studies to upregulate antioxidant enzymes (e.g., SOD and GPx) and inhibit ROS levels, as well as alleviate mitochondrial oxidative damage [37, 38, 39, 40]. This is highly consistent with the downstream effects of the Keap1/Nrf2 pathway, suggesting that these components may participate in regulating antioxidant responses with Nrf2 as the key node, collectively intervening in oxidative stress, a core pathological link of PD [37, 38, 39, 40]. Based on prior research, the active components of this formula and their intervention in PD likely target the alleviation of oxidative stress, providing significant evidence for its role in therapy.

Keap1 functions as a negative regulator of Nrf2 by tethering it within the cytoplasmic compartment through its direct interaction with the ETGE and DLG motifs of Nrf2. This interaction effectively links Nrf2 to the Cul3 ubiquitin ligase complex, thereby facilitating its ubiquitination and subsequent degradation via the proteasomal pathway [15, 42]. As a continuous degradation mechanism, this process ensures that Nrf2 is not activated in nonstress conditions, preventing resource waste and abnormal signal amplification [15, 42]. Under oxidative stress conditions, regulation of the Keap1–Nrf2 pathway involves oxidation of several cysteine residues, which are essential for responding to oxidative stress. Their synergistic action disrupts the interaction between Keap1 and Cullin3, resulting in alterations to the conformation of Keap1, which facilitates the release of Nrf2 from the Keap1 complex [43]. The inhibited degradation of Nrf2 allows it to translocate into the nucleus, where it attaches to antioxidant response elements (ARE) and promotes the synthesis of antioxidant enzymes, for example, HO‐1 [15].

In this study, to clarify whether BYQZF exerts neuroprotective roles via the Keap1/Nrf2/HO‐1 pathway, we conducted a comprehensive assessment at multiple levels, including gene expression, protein expression, signal axis activation, and functional dependency validation. qPCR results showed that in the PD model induced by MPTP, the transcriptional expression of Nrf2 was significantly downregulated, while Keap1 was significantly upregulated, indicating that the pathway was suppressed in the pathological state. After BYQZF intervention, the Nrf2 mRNA expression significantly increased, while the Keap1 expression exhibited a noticeable negative regulatory trend. This suggests that BYQZF may relieve pathway suppression by inhibiting Keap1 and promoting Nrf2 expression, thereby activating downstream antioxidant responses and laying the foundation for subsequent protein‐level signal amplification and antioxidant enzyme expression. The qPCR results provide the first evidence of signal axis activation, demonstrating the “upstream action” feature of the TCM formula. At the protein level, Western blot results further confirmed the transcriptional trends and activation of the antioxidant network. Compared to the model group, BYQZF significantly upregulated Nrf2 protein expression and simultaneously downregulated Keap1. Additionally, the expression of downstream antioxidant proteins, including HO‐1 and NQO1, was significantly increased, indicating successful activation of the signal axis and effective restoration of the cell's antioxidant network. These findings not only confirm the activation effect of BYQZF on the Nrf2 pathway but also demonstrate that its antioxidant effects are based on a multilevel transcriptional‐translational regulatory mechanism.

We used Co‐IP experiments at the protein interaction level to evaluate the binding relationship between Nrf2 and its cytoplasmic inhibitor Keap1, and attempted to construct a mechanistic chain of the Nrf2 pathway from “release of inhibition” to “transcriptional activation.” After BYQZF intervention, the interaction between Nrf2 and Keap1 was significantly weakened, indicating that Nrf2 was dissociated from Keap1 and escaped Keap1‐mediated degradation, which provided the necessary conditions for nuclear translocation and transcriptional activity. To further validate the above experimental results, we constructed Nrf2 gene knockdown stable cell lines in SH‐SY5Y cells. Under MPP^+^‐treated damage, cells in the control siRNA group showed significant mitochondrial dysfunction (ATP reduction), enhanced oxidative stress (decreased CAT, increased GSSG), and increased cell apoptosis. However, after BYQZF intervention, these indicators were significantly improved, suggesting that BYQZF could effectively alleviate the cytotoxicity induced by MPP^+^. In contrast, in the Nrf2 siRNA knockdown group, where Nrf2 expression was stably suppressed, the efficacy of BYQZF was notably reduced. Its restorative effect on cell viability, ability to enhance ATP levels, and anti‐apoptotic and antioxidant abilities (such as inhibition of apoptosis, recovery of CAT, and reduction of GSSG) were all significantly weakened. Particularly in flow cytometry detection, BYQZF failed to effectively reduce the apoptosis rate in the Nrf2‐deficient background, further highlighting the core role of the Nrf2 pathway in its anti‐apoptotic mechanism. The reverse intervention experiments provided functional evidence that the neuroprotective functions of BYQZF highly depend on the integrity and activation of Nrf2. That is, Nrf2 deficiency blocks its transcriptional response to oxidative stress signals and the regulation of downstream anti‐apoptotic networks. This result not only corroborates the evidence of Nrf2 activation in immunoprecipitation and WB experiments but also causally confirms that the Keap1/Nrf2/HO‐1 axis is the key mediating pathway through which BYQZF exerts its neuroprotective effects. BYQZF promotes the dissociation of Nrf2 from the Keap1 complex through upstream regulatory events, activates the expression of downstream antioxidant factors, and ultimately achieves a multistep linkage from Nrf2 activation to antioxidant enzyme expression, thereby exerting significant neuroprotective effects (Graphical Abstract). Our findings not only provide molecular‐level evidence, but also offer theoretical support and potential target identification for its precise application in targeting the Nrf2/Keap1/HO‐1 pathway in PD.

Although this study confirmed the key role of Nrf2 in mediating the antioxidant effect of BYQZF through siRNA knockdown experiments in vitro, providing solid evidence for our conclusion, we must acknowledge the limitations of its existence. The main limitation is that the evidence of Nrf2 functional dependence provided by this study mainly comes from cellular models. Although siRNA knockdown is an effective and specific means, in vivo verification is indispensable to ultimately establish the causal role of Nrf2 in the overall pharmacological effects of BYQZF. Therefore, using the Nrf2 knockout mouse model for experimental verification will be a crucial step in future research, which will directly and irrefutably reveal whether the Nrf2 pathway is the only or main way for BYQZF to exert its antioxidant and protective effects under a complex physiological environment.

As previously indicated, there are several shortcomings in the drug treatment options for PD. While drug treatments can alleviate motor symptoms in the short term, long‐term use is often associated with multiple side effects. Most treatment options focus only on dopamine metabolism regulation, which fails to effectively intervene in the essential mechanisms of disease progression—oxidative stress, mitochondrial damage, and neuronal apoptosis. However, BYQZF can alleviate motor dysfunction and neural damage in PD model mice by activating the Keap1/Nrf2/HO‐1 antioxidant pathway. More importantly, multiple active ingredients in the compound (such as berberine, rehmannioside, and mangiferin) act synergistically on the oxidative‐inflammation‐apoptosis network in the nervous system, holding the potential for neuroprotection through multitarget intervention rather than single‐point repair. In the modern PD treatment system, BYQZF not only serves as an adjunct but also complements and breaks through traditional methods at the mechanistic level. Although we have constructed a complete causal chain from mechanism validation to functional improvement and further expanded the modern pharmacological basis for traditional Chinese medicine compound intervention in PD, it should be noted that the research mainly focuses on the verification of gene and protein expression in the core pathway, without high‐throughput omics screening such as transcriptomics or proteomics. Future research could further explore other potential action networks of BYQZF, providing richer mechanistic evidence for its clinical precision application.

## Author Contributions

Cong Gai and Huimin Zhu designed and planned this paper. Huimin Zhu, Jing Feng, and Zijian Liu combined the literature and drafted the article. Die Hu, Xia Li, Xueying Zhu and Zhenyu Guo revised the manuscript. All authors contributed to the article and approved the submitted version.

## Funding

This work was supported by the National Natural Science Foundation of China (No. 82405153 and 82104644), Fundamental Research Funds for the Central Universities (No. 2022‐JYB‐XJSJJ003) and National Administration of Traditional Chinese Medicine (No. zyyzdxk‐2023262).

## Ethics Statement

All animal experiments were carried out in accordance with the “Guidelines for Care and Use of Laboratory Animals” (NIH Publication No. 85‐23, revised 1996) published by the US National Institutes of Health. All procedures were approved by the Animal Care Committee of Beijing University of Chinese Medicine (Approval Code: BUCM‐2024051602‐2130).

## Conflicts of Interest

The authors declare no conflicts of interest.

## Supporting information


**Figure S1:** The morphology and structure of the vital organs in different groups were observed by H&E staining BYQZF‐L (5 g crude drug/kg) and BYQZF‐H (15 g crude drug/kg) were administered intragastrically every day for 14 days. The administration volume was 10 mL/kg, and the concentration of the drug solution was 0.5 g/mL and 1.5 g/mL, respectively. Madopar (62.5 mg/kg) were administered intragastrically every day for 14 days. Control (saline) or MPTP (20 mg/kg of free base) was injected intraperitoneally during 5 consecutive days before decoction treatment. Scale bars are 100 μm.
**Figure S2:** The levels of ALT, AST, CREA, LDH, CK, IL‐6, TNF‐a, and IL‐1β in different groups. BYQZF‐L (5 g crude drug/kg) and BYQZF‐H (15 g crude drug/kg) were administered intragastrically every day for 14 days. The administration volume was 10 mL/kg, and the concentration of the drug solution was 0.5 g/mL and 1.5 g/mL, respectively. Madopar (62.5 mg/kg) were administered intragastrically every day for 14 days. Control (saline) or MPTP (20 mg/kg of free base) was injected intraperitoneally during 5 consecutive days before decoction treatment. 1: Control; 2: MPTP; 3: BYQZF‐L; 4: BYQZF‐H; 5: MPTP + Madopar; 6: MPTP + BYQZF‐L. Data are expressed as mean ± SEM (*N* = 6).
**Figure S3:** Typical mass spectrum chromatograms of BYQZF. Negative ion mode base peak chromatogram (BPC) of BYQZF; 1: L‐Glutamic acid; 2: L‐beta‐aspartyl‐L‐aspartic acid; 3: Hyperin; 4: Rehmannioside A; 5: Ajugol; 6: 8‐Epiloganic acid; 7: Neomangiferin; 8: Aucubin; 9: Mangiferol; 10: Isomangiferin; 11: Verbasoside; 12: Isoacteoside; 13: 3‐Hydroxyflavone; 14: Timosaponin AIII;15: Bocinic acid. (B) Positive ion mode BPC of BYQZF. 1: Manninotriose; 2: Inositol; 3: Uracil; 4: alpha‐Terpinyl acetate; 5: Linonin.
**Figure S4:** Plasma concentration‐time distribution of major components after oral administration of BYQZF (*n* = 6).
**Figure S5:** All Western blot bands.

**Table S1:** Qualitative and quantitative results of BYQZF components.

**Table S2:** Pharmacokinetic parameters of 10 major components after oral administration of BYQZF to mice (*N* = 6).

## Data Availability

The data that support the findings of this study are available from the corresponding author upon reasonable request.
