# Supplementary material for: Da‐Bu‐Yin‐Wan and Qian‐Zheng‐San Alleviate Parkinson's Disease by Activating the Keap1/Nrf2/HO‐1 Pathway to Positively Regulate Oxidative Stress
Source: CNS Neurosci Ther. 2025 Dec 9;31(12):e70681. doi: 10.1002/cns.70681 (PMC12686966; doi:10.1002/cns.70681)
Supplement: Supplementary file 1 — Figure S1: The morphology and structure of the vital organs in different groups were observed by H&E staining BYQZF‐L (5 g crude drug/kg) and BYQZF‐H (15 g crude drug/kg) were administered intragastrically every day for 14 days. The administration volume was 10 mL/kg, and the concentration of the drug solution was 0.5 g/mL and 1.5 g/mL, respectively. Madopar (62.5 mg/kg) were administered intragastrically every day for 14 days. Control (saline) or MPTP (20 mg/kg of free base) was injected intraperitoneally during 5 consecutive days before decoction treatment. Scale bars are 100 μm. Figure S2: The levels of ALT, AST, CREA, LDH, CK, IL‐6, TNF‐a, and IL‐1β in different groups. BYQZF‐L (5 g crude drug/kg) and BYQZF‐H (15 g crude drug/kg) were administered intragastrically every day for 14 days. The administration volume was 10 mL/kg, and the concentration of the drug solution was 0.5 g/mL and 1.5 g/mL, respectively. Madopar (62.5 mg/kg) were administered intragastrically every day for 14 days. Control (saline) or MPTP (20 mg/kg of free base) was injected intraperitoneally during 5 consecutive days before decoction treatment. 1: Control; 2: MPTP; 3: BYQZF‐L; 4: BYQZF‐H; 5: MPTP + Madopar; 6: MPTP + BYQZF‐L. Data are expressed as mean ± SEM (N = 6). Figure S3: Typical mass spectrum chromatograms of BYQZF. Negative ion mode base peak chromatogram (BPC) of BYQZF; 1: L‐Glutamic acid; 2: L‐beta‐aspartyl‐L‐aspartic acid; 3: Hyperin; 4: Rehmannioside A; 5: Ajugol; 6: 8‐Epiloganic acid; 7: Neomangiferin; 8: Aucubin; 9: Mangiferol; 10: Isomangiferin; 11: Verbasoside; 12: Isoacteoside; 13: 3‐Hydroxyflavone; 14: Timosaponin AIII;15: Bocinic acid. (B) Positive ion mode BPC of BYQZF. 1: Manninotriose; 2: Inositol; 3: Uracil; 4: alpha‐Terpinyl acetate; 5: Linonin. Figure S4: Plasma concentration‐time distribution of major components after oral administration of BYQZF (n = 6). Figure S5: All Western blot bands. [file CNS-31-e70681-s003.doc]

**Supplementary Information**

**Da-Bu-Yin-Wan and Qian-Zheng-San alleviates Parkinson's disease by activating the Keap1/Nrf2/HO-1 pathway to positively regulate oxidative stress**

**Huimin Zhu ^a^, Zijian Liu ^b^, Jing Feng ^a^, Die Hu ^a^, Xia Li ^a^, Zhenyu Guo ^a^, Xueying Zhu ^a^, Cong Gai ^a,*^**

a School of Chinese Medicine, Beijing University of Chinese Medicine, Beijing, 102488, China.

b Graduate School of the First Clinical Medical College, Beijing University of Chinese Medicine, Beijing, 100007, China

***Corresponding Authors:**

Pro. Cong Gai; School of Chinese Medicine, Beijing University of Chinese Medicine, 11 North Third Ring East Road, Chaoyang District, Beijing 102488, China; E-mail: [gaicong9046@163.com;](mailto:gaicong9046@163.com;) Tel/Fax: +86-10-5391 1430


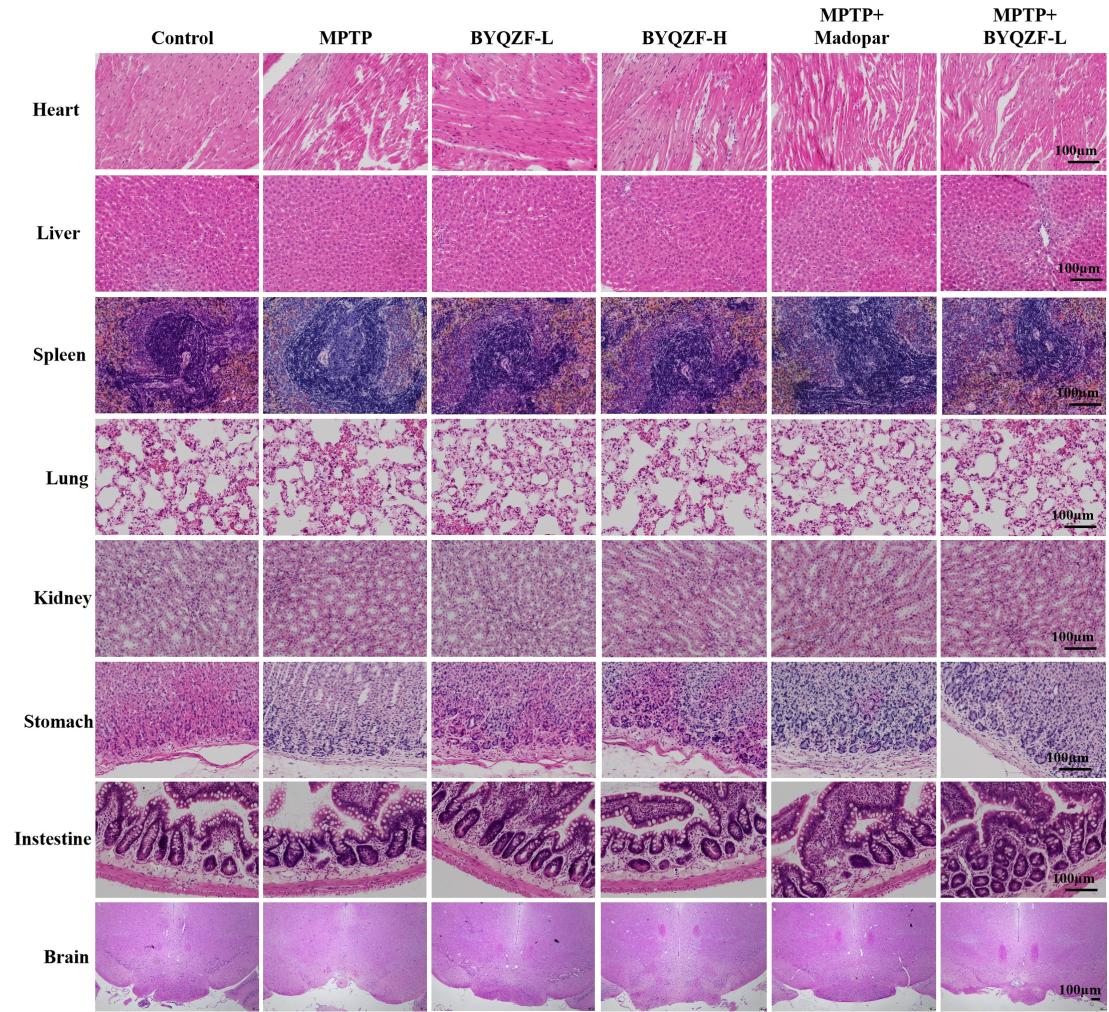


**Fig.S1. The morphology and structure of the vital organs in different groups were observed by H&E staining.**

BYQZF-L (5g crude drug/kg) and BYQZF-H (15g crude drug/kg) were administered intragastrically everyday for 14 days. The administration volume was 10 ml/kg, and the concentration of the drug solution was 0.5 g/ml and 1.5 g/ml, respectively. Madopar (62.5mg/kg) were administered intragastrically everyday for 14 days. Control (saline) or MPTP (20 mg/kg of free base) was injected intraperitoneally during 5 consecutive days before decoction treatment. Scale bars are 100 µm.


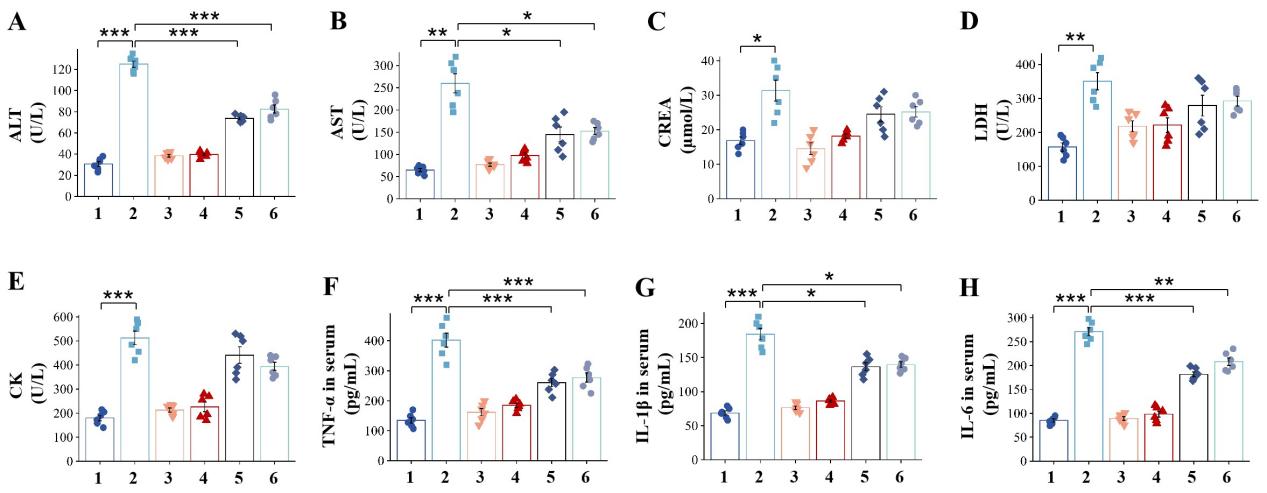


**Fig.S2. The levels of ALT, AST, CREA, LDH, CK, IL-6, TNF-α and IL-1β in different groups.**

BYQZF-L (5g crude drug /kg) and BYQZF-H (15g crude drug/kg) were administered intragastrically everyday for 14 days. The administration volume was 10 ml/kg, and the concentration of the drug solution was 0.5 g/ml and 1.5 g/ml, respectively. Madopar (62.5mg/kg) were administered intragastrically everyday for 14 days. Control (saline) or MPTP (20 mg/kg of free base) was injected intraperitoneally during 5 consecutive days before decoction treatment. 1:Control; 2: MPTP; 3: BYQZF-L; 4:BYQZF-H; 5: MPTP+Madopar; 6: MPTP+BYQZF-L. Data are expressed as mean±SEM (*N*=6).

*
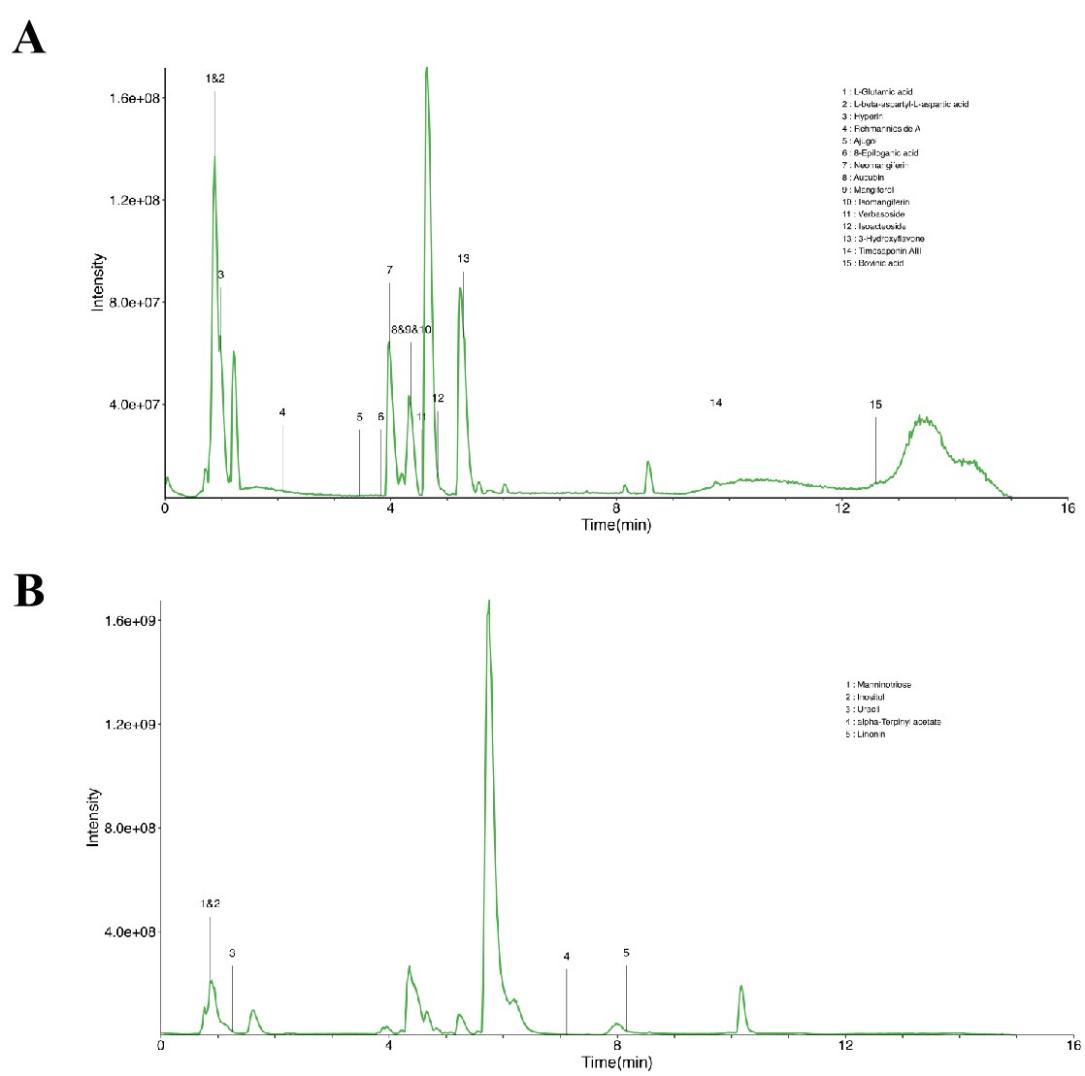
*

**FigS3. Typical mass spectrum chromatograms of BYQZF.**

(A) Negative ion mode base peak chromatogram (BPC) of BYQZF; 1: L-Glutamic acid; 2: L-beta-aspartyl-L-aspartic acid; 3: Hyperin; 4: Rehmannioside A; 5: Ajugol; 6: 8-Epiloganic acid; 7: Neomangiferin; 8: Aucubin; 9: Mangiferol; 10: Isomangiferin; 11: Verbasoside; 12: Isoacteoside; 13: 3-Hydroxyflavone; 14: Timosaponin AⅢ;15: Bocinic acid. (B) Positive ion mode BPC of BYQZF. 1: Manninotriose; 2: Inositol; 3: Uracil; 4:alpha-Terpinyl acetate; 5: Linonin


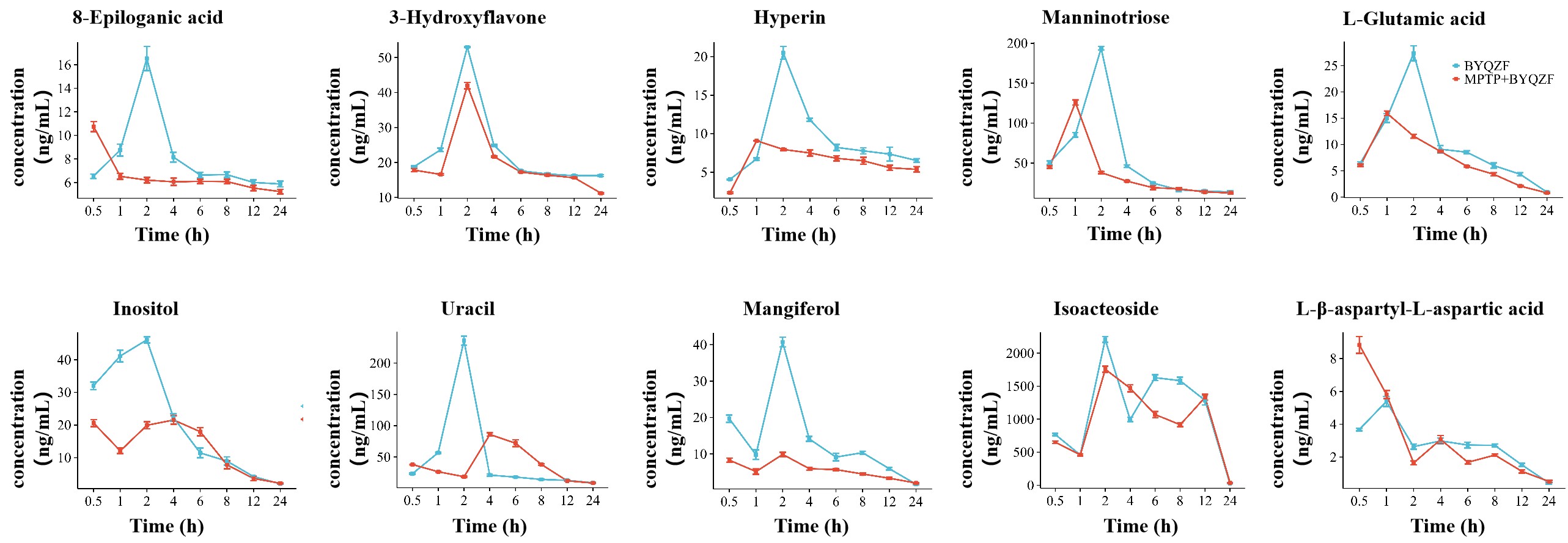


**FigS4. Plasma concentration-time distribution of major components after oral administration of BYQZF (mean±SD, n=6).**

**
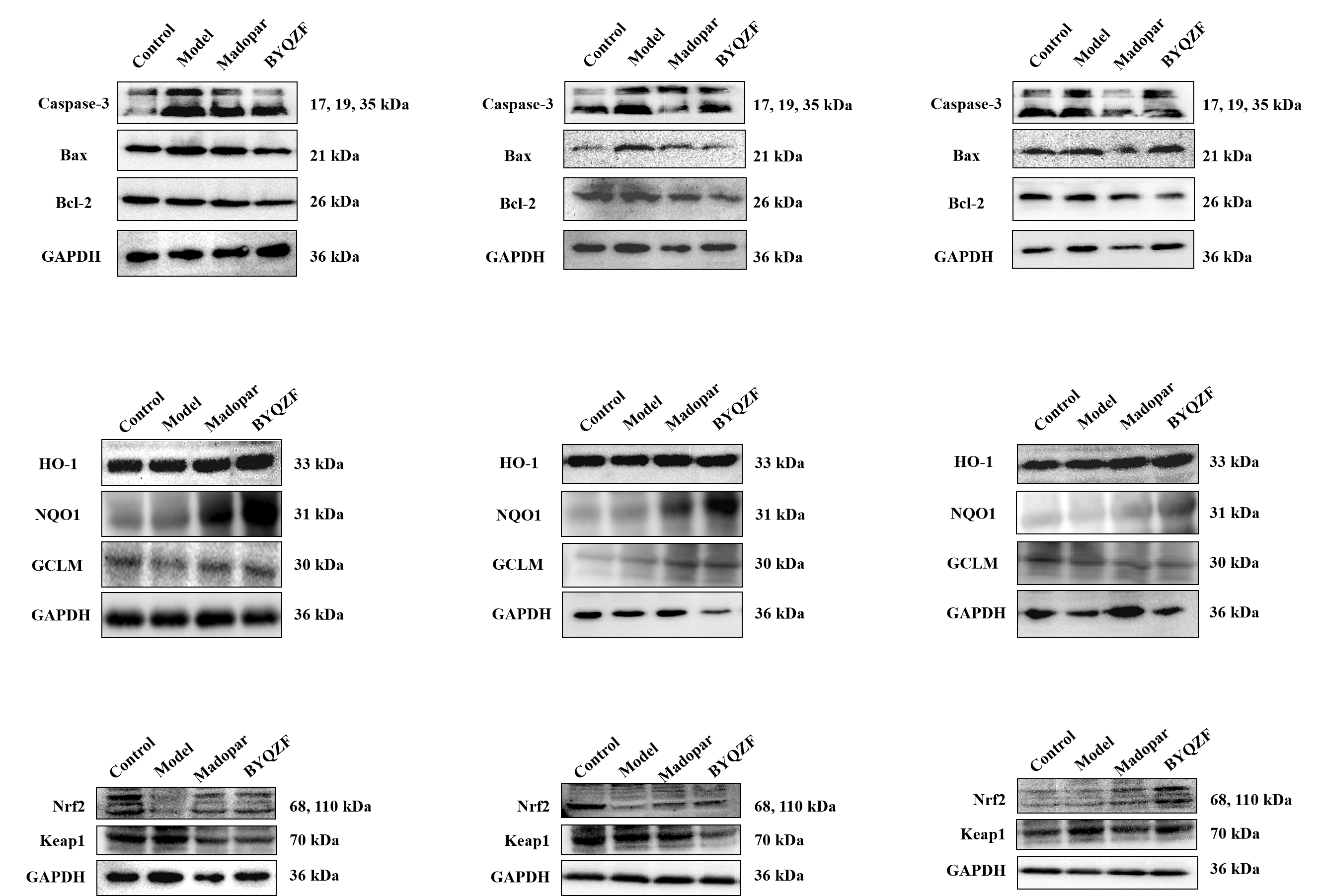
**

**FigS5. All Western blot bands.**

**TableS1 qualitative and quantitative results of BYQZF components.**

**TableS2 Pharmacokinetic parameters of 10 major components after oral administration of BYQZF to mice (n=6).**
